# Genome-wide association study and haplotype analysis reveal novel candidate genes for resistance to powdery mildew in soybean

**DOI:** 10.3389/fpls.2024.1369650

**Published:** 2024-03-27

**Authors:** Guoqiang Liu, Yuan Fang, Xueling Liu, Jiacan Jiang, Guangquan Ding, Yongzhen Wang, Xueqian Zhao, Xiaomin Xu, Mengshi Liu, Yingxiang Wang, Cunyi Yang

**Affiliations:** ^1^ Guangdong Provincial Key Laboratory of Plant Molecular Breeding, College of Agriculture, South China Agricultural University, Guangzhou, China; ^2^ Key Laboratory for Enhancing Resource Use Efficiency of Crops in South China, Ministry of Agriculture and Rural Affairs, South China Agricultural University, Guangzhou, China; ^3^ Guangdong Laboratory for Lingnan Modern Agriculture, Guangzhou, China; ^4^ Guangdong Provincial Key Laboratory of Protein Function and Regulation in Agricultural Organisms, College of Life Sciences, South China Agricultural University, Guangzhou, China

**Keywords:** GWAS, PMD, haplotypes, qRT-PCR, EMS mutations, NLR

## Abstract

Powdery mildew disease (PMD) is caused by the obligate biotrophic fungus *Microsphaera diffusa* Cooke & Peck (*M. diffusa*) and results in significant yield losses in soybean (*Glycine max* (L.) Merr.) crops. By identifying disease-resistant genes and breeding soybean accessions with enhanced resistance, we can effectively mitigate the detrimental impact of PMD on soybeans. We analyzed PMD resistance in a diversity panel of 315 soybean accessions in two locations over 3 years, and candidate genes associated with PMD resistance were identified through genome-wide association studies (GWAS), haplotype analysis, qRT-PCR, and EMS mutant analysis. Based on the GWAS approach, we identified a region on chromosome 16 (Chr16) in which 21 genes form a gene cluster that is highly correlated with PMD resistance. In order to validate and refine these findings, we conducted haplotype analysis of 21 candidate genes and indicated there are single nucleotide polymorphisms (SNPs) and insertion-deletions (InDels) variations of *Glyma.16G214000, Glyma.16G214200, Glyma.16G215100* and *Glyma.16G215300* within the coding and promoter regions that exhibit a strong association with resistance against PMD. Subsequent structural analysis of candidate genes within this cluster revealed that in 315 accessions, the majority of accessions exhibited resistance to PMD when *Glyma.16G214300, Glyma.16G214800 *and *Glyma.16G215000* were complete; however, they demonstrated susceptibility to PMD when these genes were incomplete. Quantitative real-time PCR assays (qRT-PCR) of possible candidate genes showed that 14 candidate genes (*Glyma.16G213700, Glyma.16G213800, Glyma.16G213900, Glyma.16G214000, Glyma.16G214200, Glyma.16G214300, Glyma.16G214500, Glyma.16G214585, Glyma.16G214669, Glyma.16G214700, Glyma.16G214800, Glyma.16G215000, Glyma.16G215100* and *Glyma.16G215300*) were involved in PMD resistance. Finally, we evaluated the PMD resistance of mutant lines from the Williams 82 EMS mutations library, which revealed that mutants of *Glyma.16G214000, Glyma.16G214200, Glyma.16G214300, Glyma.16G214800, Glyma.16G215000, Glyma.16G215100* and *Glyma.16G215300*, exhibited sensitivity to PMD. Combined with the analysis results of GWAS, haplotypes, qRT-PCR and mutants, the genes *Glyma.16G214000, Glyma.16G214200, Glyma.16G214300, Glyma.16G214800, Glyma.16G215000, Glyma.16G215100* and *Glyma.16G215300* were identified as highly correlated with PMD resistance. The candidate genes identified above are all NLR family genes, and these discoveries deepen our understanding of the molecular basis of PMD resistance in soybeans and will be useful for guiding breeding strategies.

## 1 Introduction

Soybean [*Glycine max* (L.) Merr.] is a leguminous crop that provides approximately 71% of plant-based protein and 29% of oil globally (Lin et al., 2022) and is an important source of animal and aquaculture feed (Adak and Kibritci, 2016). However, the proportion of soybean yield loss due to disease is increasing (Lin et al., 2022). Powdery mildew disease (PMD) is a common soybean disease caused by the fungus *Microsphaera diffusa* (*M. diffusa*) particularly in temperatures ranging from 15°C to 30°C. When the temperature is below 15°C or above 30°C, PMD infections will be reduced (Alves et al., 2009). It is easily detected on seeds, stems, leaves, and roots as white and powdery patches (Ramalingam et al., 2020) that result in defoliation, chlorosis veinal, necrosis, or mixtures of several symptoms (Grau, 2006). PMD causes yield reductions of up to 35% in susceptible soybean accessions (Dunleavy, 1980; Phillips, 1984) and is an epidemic disease in Australia (McTaggart et al., 2012), Canada (Takamatsu et al., 2002), Peru (Takamatsu et al., 2002), Puerto Rico (Takamatsu et al., 2002), Venezuela (Takamatsu et al., 2002), Brazil (Goncalves et al., 2002), Asia (Li M. W. et al., 2020), northeast India (Baiswar et al., 2016), and the United States (Grau, 2006). Despite the global importance of PMD as a soybean disease, the molecular basis of resistance and susceptibility to it remain largely uncharacterized.

Several PMD resistance loci in multiple soybean accessions have been mapped to the Chr16, including *Rmd_V97-3000* (Wang et al., 2013), *Rmd_PI243540* (Kang and Mian, 2010), *Rmd_PI567301B* (Jun et al., 2012), *Rmd_B3* (Jiang et al., 2019), *Rmd_B13* (Jiang et al., 2019), and *Rmd_ZH24* (Zhou et al., 2022). Mapping indicates they overlap or partially overlap each other suggesting that they could be tightly linked loci or one gene with different alleles. *Rmd_B13* was mapped to a genomic region containing 17 disease resistance (*R*)-like genes (Jiang et al., 2019), and *Rmd_ZH24* to an interval with 4 disease *R*-like genes (Zhou et al., 2022). Recently, the first soybean PMD resistance gene, *GmRmd1*, has been cloned through a combination of multiple methods, including a genome-wide association study from 467 soybean accessions, map-based cloning of 471 F_8_ recombinant inbred lines derived from Guizao1 (susceptible) × B13 (resistant), and denovo assembly of the Guizao1 and B13 draft genomes using single-molecule long-read sequencing technology that can explore SVs in the *GmRmd1* region (Xian et al., 2022). By contrast, genes associated with PMD resistance have been extensively studied in other plants. In *Arabidopsis thaliana*, the *RESISTANCE TO POWDERY MILDEW 8.2* (*RPW8.2*) gene encodes phosphatase type 2C (PAPP2C), which negatively regulates salicylic acid (SA)-dependent basal defense against PMD (Wang et al., 2012). The barley *Mla* locus contains *Mla1* (Zhou et al., 2001), *Mla12* (Shen et al., 2003), and *Mla13* (Lu et al., 2016), which are nucleotide-binding and leucine-rich repeat (NLR) family proteins that recognize avirulence (AVR) proteins from the PMD fungus *Blumeria graminis* f. sp. *hordei*. In wheat, nearly 70 PMD resistance loci have been identified (Muller et al., 2022), but only a few genes have been cloned, including *Pm3* (Yahiaoui et al., 2004), *Pm8* (Hurni et al., 2013), *Pm17* (Singh et al., 2018), *Pm21* (Singh et al., 2018; Xing et al., 2018), *Pm60* (Zou et al., 2017), *Pm5* (Xie et al., 2020), and *Pm41* (Li M. et al., 2020). They all encode NLR family proteins.

Genome-wide association study (GWAS) is a genetic marker detection technique that has evolved into a pivotal method for investigating the genetics of intricate diseases (Burghardt et al., 2017). Compared to conventional linkage analysis, GWAS can significantly enhance the precision and accuracy of marker–phenotype associations (Yano et al., 2016; Cortes et al., 2021). Currently, GWAS has been successfully employed in elucidating the genetic basis underlying soybean PMD resistance. In 2020, the first GWAS analysis of soybean PMD resistance was used in gene mapping research, and this study obtained 30,510 high-quality SNP loci from 331 soybean accessions for association analysis resulting in the identification of *Glyma.16g210800*, *Glyma.16g211000*, and *Glyma.16g211400* as important candidate genes for disease resistance (Hu, 2021). In addition, Xian utilized GWAS to classify the genetic polymorphisms associated with PMD resistance in soybeans, identified a single region associated with PMD resistance based on 2,176,969 SNPs in 467 soybean accessions, and finally identified *GmRmd1* as the PMD resistance gene in this region (Xian et al., 2022). Another study identified seven SNPs significantly associated with resistance to PMD through GWAS, and combined with differential expression levels, three candidate genes for *Rmd_ZDD00359* were determined: *Glyma.16G210800*, *Glyma.16G212300*, and *Glyma.16G213900* (Sang et al., 2023). In recent years, this method has been utilized to identify numerous QTLs and genes governing crucial disease-resistant traits in soybean. For instance, through GWAS analysis, a total of 36,976 SNP markers associated with resistance to soybean cyst nematode (SCN) *Heterodera glycines* (HG) Ichinohe 0 type and *Heterodera glycines* Ichinohe 1.2.3.5.7 type were identified across a diverse panel of 440 soybean accessions, and a total of 19 associated signals were detected and significantly correlated with the resistance of two types of HG (Han et al., 2015; Cortes et al., 2021). A GWAS analysis was performed on a total of 330 soybean accessions, resulting in the identification of 25,179 SNPs and the discovery of eight genomic regions significantly associated with resistance to soybean white mold (Boudhrioua et al., 2020). Utilizing a whole-genome association mapping approach, 800 soybean accessions were employed to identify genomic regions associated with resistance to phytophthora root and stem rot, and 16 SNP markers located on chromosomes 3, 13, and 19 exhibited a significant correlation with resistance of soybeans to this disease (Schneider et al., 2016). Therefore, GWAS holds significant potential for enhancing disease resistance in soybean breeding.

The objective of this article is to investigate the role of NLR gene clusters in conferring soybean resistance against PMD, thereby establishing a fundamental understanding of plant disease resistance mechanisms and providing valuable insights for breeding PMD-resistant soybean accessions.

## 2 Materials and methods

### 2.1 Plant materials and evaluation of PMD resistance

Williams 82 (W82) and Huaxia 3 (HX3) were provided by South China Agricultural University. The 315 soybean accessions used for this study were obtained from soybean research institutions in China (**Supplementary Table 1**). Accessions were planted in December in field sites located in Guangzhou and Hainan, and these were used for disease evaluation and genetic analysis.

In December 2017, accessions were sown in the field of Hainan Experimental Station, China. Disease resistance was evaluated in February 2018, and the data were recorded as Y2018. In December 2018 and December 2019, accessions were grown in the field at South China Agricultural University, Guangzhou. PMD resistance was evaluated in March 2019 and March 2020, and the data were recorded as Y2019 and Y2020, respectively. All plants were scored and counted for PMD resistance (**Supplementary Table 1**). All plants were challenged with *M. diffusa* as described previously (Jiang et al., 2019). Each plant was inoculated with spores of *M. diffusa* at stage V1 by brushing with PMD-infected leaves of susceptible plants maintained in the greenhouse, as described by Kang and Mian (2010). Three weeks after inoculation, PMD incidence was evaluated for the 315 soybean accessions. Accessions were scored for PMD resistance using a scale. A plant with no PMD colonies on any leaf was regarded as a resistant (R) line and scored “L0,” while a plant with one or more PMD colonies on its leaves was rated as a susceptible (S) line, depending on disease severity, from L1 grade to L5 (**Supplementary Table 1**) (Li et al., 2016).

### 2.2 DNA extraction and analysis

The sodium dodecyl sulfonate (SDS) method was used to extract DNA from GWAS populations from young unfolded trifoliolate leaves at stage V2 (second trifoliolate) (Perry, 2004). A minimum of 30 ng/µl of DNA, with an OD260/280 of 1.8–2.0, was used for resequencing, and greater than 2 µg of DNA was used to generate small fragment libraries for pair-end sequencing.

### 2.3 Genome mapping and detection of SNPs and InDels

A total of 315 soybean accessions were selected for whole-genome re-sequencing using the Illumina HiSeq X Ten platform with 150-bp pair-end reads. The average sequencing depth of all samples is 15×. A detailed sequencing information is shown in **Supplementary Table 2**. All the sequencing in this study was done by Annoroad Gene Technology Co. Ltd. (Zhejiang, China).

Version 4 of Williams 82 genome in the Phytozome database (https://phytozome-next.jgi.doe.gov/info/Gmax_Wm82_a4_v1) was used as the reference for data analysis. Read quality control to remove low-quality reads and adapter sequences was done using Fastp (version 0.23.2) (Chen et al., 2018) with default parameters. Clean reads from each sample were aligned to the reference genome using BWA-MEM 2 (release 2.2.1) (Vasimuddin et al., 2019), and then, mapped reads were sorted using SAMtools (version 1.15.1) (Li et al., 2009). GATK4 packages (release 4.2.6.1) (Mckenna et al., 2010) were employed for variation detection and haplotype analysis. Duplicate-read marking was performed using MarkDuplicates. Variants from each sample were called using HaplotypeCaller to generate files in gVCF format. The gVCF files were merged using CombineGVCFs generating a VCF file composed of emit-all-sites information. The VCF file was filtered using the recommended GATK parameters “QUAL < 30.0 || QD < 2.0 || MQ < 40.0 || FS > 60.0 || SOR > 3.0 || MQRankSum < −12.5 || ReadPosRankSum < −8.0” and “QUAL < 30.0 || QD < 2.0 || FS > 200.0 || SOR > 10.0 || ReadPosRankSum < -20.0 || MQ < 40.0 || MQRankSum < −12.5” for SNPs and InDels, respectively. Apart from this, SNPs were filtered using the following criteria: removing SNPs as missing greater than 15% and MAF lower than 0.5% among 315 accessions.

### 2.4 Detecting significant SNPs

TASSEL (version 5.2.8.1) (Bradbury et al., 2007) was used to calculate the association of the PMD resistance phenotype with genetic polymorphisms in 315 soybean accessions by applying a mixed linear model (MLM). In this study, admixture software was employed to estimate the population structure matrix (Q). When K = 11, the CV error was the minimum value at 0.54741. TASSEL5 software was used to calculate the kinship matrix (K) with the parameter-method Centered_IBS. As covariates, Q and K were used to control the population in the MLM analysis. As part of the TASSEL analysis, the phenotypic variation explained (PVE) was also calculated using the mixed liner model. The CMplot package (Yin et al., 2021) was used for visualizing the Manhattan p and QQ plots. The Manhattan plot shows the p-values of significant SNPs associated with PMD resistance. The QQ plot shows the relationship between theory and expectation. SnpEff (Cingolani et al., 2012) was used for gene annotation of heterotopic points according to the physical location and structure of genes throughout the genome.

### 2.5 Analysis of linkage disequilibrium and haplotype blocks

LD analysis measures the nonrandom association of pairs of SNPs. TASSEL (version 5.2.8.1) (Bradbury et al., 2007) software calculates the square (R2) of the allele frequency correlation of SNP. The core SNP set derived after suitable filtering was used for calculating linkage disequilibrium (LD) patterns and the structure of haplotype blocks of SNPs by LDBlockShow (Dong et al., 2020). Data sets (r2 and D′) of pairwise LD measurement calculations showed LD levels of SNPs.

### 2.6 Haplotype analysis of candidate genes

The genotype code types and physical position in the genome of the candidate locus were obtained from genotype-calling VCF format files. Unsupervised clustering was applied to three genotype codes consisting of reference homozygote, alternative homozygote, and missing by Cluster3.0 software (Hoon et al., 2004).

### 2.7 Evaluation of the expression patterns of 21 candidate genes

V1 stage W82 and HX3 plants were infected with an *M. diffusa* spore suspension containing 1 × 10^5^ cfu/ml and maintained in a growth chamber at 23°C, 75% relative humidity, with a 16-h light/8-h dark photoperiod to characterize the expression of 21 candidate genes. The experiments included three replicates. Leaves were sampled at 0, 6, 12, 24, 48, and 72 h after inoculation and kept at −80°C. Total RNA was extracted using the HiPure Plant RNA Mini Kit (Magen, Guangzhou, Guangdong, China), and 1 mg of total RNA was reverse transcribed to produce first-strand cDNA using the *Evo M-MLV* RT Kit with gDNA Clean for qRT-PCR (Accurate Biology, Guangzhou, Guangdong, China). Candidate genes in the target region were predicted in SoyBase using the Wm82.a4.v1 reference genome. Quantitative real-time polymerase chain reaction (qRT-PCR) was performed to obtain the expression profiles of candidate genes using primers designed with Primer Premier 5.0 (Premier, Vancouver, Canada). The housekeeping gene *Actin* was used as a control. The specific primers for 21 candidate genes are listed in **Supplementary Table 6**. The qRT-PCR was performed with a CFX96 Real-Time PCR Detection System (Bio-Rad, Hercules, CA, USA) using SYBR^®^ Green Premix *Pro Taq* HS qPCR Kit II (Accurate Biology, Guangzhou, Guangdong, China). All reactions were performed in 20-µl volumes containing 1 µl of cDNA as a template. The thermal cycling conditions consisted of 94°C for 2 min, followed by 40 cycles of 95°C for 10 s, 59°C for 30 s, and 72°C for 30 s. Three independent biological repeats were used. The qRT-PCR data were evaluated using the 2^−△△CT^ method (Livak and Schmittgen, 2001).

### 2.8 The PMD resistance evaluation of mutant lines from Williams 82 EMS mutation library

The information of mutant lines from the Williams 82 EMS-induced library was extracted from this website: http://isoybean.org/ (**Supplementary Table 7**). Seeds for mutants were obtained from Song’s laboratory (Zhang et al., 2022). These mutant lines were grown in the greenhouse at South China Agricultural University, Guangzhou. The mutant lines were subjected to two generations of selfing to obtain homozygous lines of the corresponding candidate genes (**Supplementary Table 7**). Each mutant plant was inoculated with spores of *M. diffusa* at stage V1. After 2 weeks of inoculation, the *M. diffusa* of the mutant leaves was observed and photographed, and these leaves were stored at −20°C for DNA extraction and sequencing.

### 2.9 Phylogenetic analysis

Candidate gene protein sequence from the reference genome annotation Wm82.a4.v1 version was used to blast the RefSeq Select proteins database (https://www.ncbi.nlm.nih.gov/refseq/refseq_select/). The top 100 sequences with the highest similarity were selected for complete multiple sequence alignment. The phylogenetic tree was constructed using the NJ method in MEGA X (Kumar et al., 2018) with 1,000 bootstrap replicates.

## 3 Results

### 3.1 Evaluation of soybean germplasm for resistance to PMD

**Figure 1:**
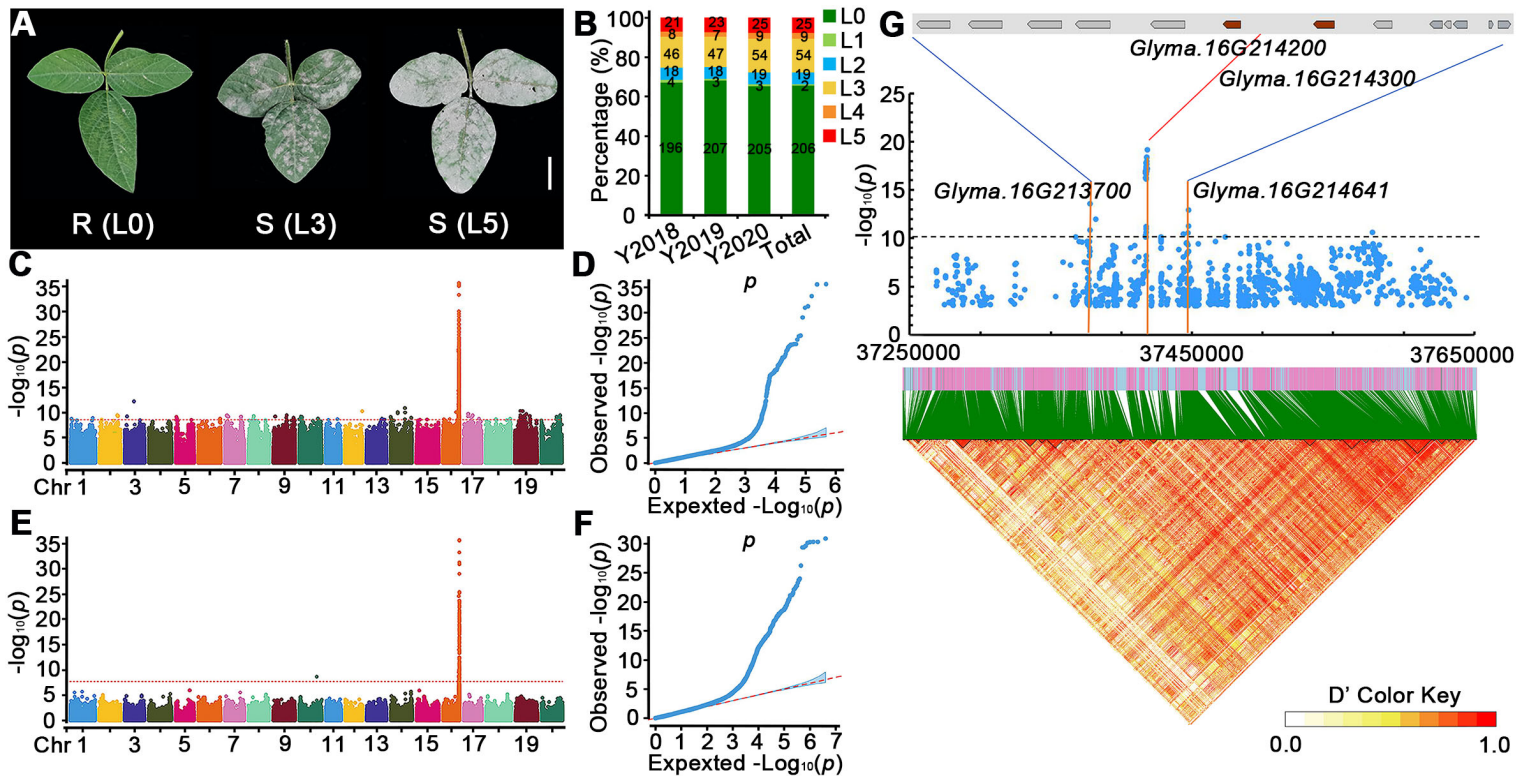
Genome-wide association analysis of PMD resistance genes in the 315 soybean accessions. **(A)** Phenotypic traits of soybean accessions after 20 days of *M. diffusa* infection. R, resistant; S, susceptible. Scale bar = 2 cm. **(B)** Percentages of PMD-resistant levels of 315 accessions in different regions and years. L0 represents resistant to PMD, and L1–L5 represent susceptible levels to PMD increasingly in **(A, B)**. Y2018: Hainan, February 2018; Y2019: SCAU field, March 2019; Y2020: SCAU field, March 2020. **(C–F)** GWAS scan for PMD using re-sequencing data of 315 accessions grown in spring from different regions and years; **(C, D)** represent Manhattan plots of significant SNPs associated with PMD and quantile–quantile plots for PMD, respectively. **(E, F)** represent Manhattan plots of significant InDels associated with PMD and quantile–quantile plots for PMD, respectively. The GWAS results are presented as negative log10 p-values against position on each of the 20 chromosomes. Horizontal red dashed lines indicate the genome-wide significant threshold. **(G)** Local Manhattan plot (top) and linkage disequilibrium heatmap (bottom) surrounding the peak on Chr16. Two genes of brown color (*Glyma.16G214200* and *Glyma.16G214300*) are flanked on both sides by the most significant SNPs (top panel). A local Manhattan plot and linkage disequilibrium heatmap of that 400-kb region are displayed in the bottom panel. The orange lines indicate the candidate region for the peak. The red line indicates the highest peak value. The blue plot indicates the nucleotide variation of the candidate genes. Horizontal black dashed lines indicate the genome-wide significant threshold. The colors in the figure from yellow to red represent the D′ value from low degree to high, which is the standardized disequilibrium coefficient between a pair of alleles.

To study PMD resistance in soybeans, we collected 315 soybean accessions from around the world and evaluated their resistance to PMD during 3 years in Hainan province (P.R. China) and an experimental field at South China Agricultural University (SCAU). We divided our screening results into five levels (L1–L5) of PMD susceptible according to the previous studies (Kang and Mian, 2010) and defined PMD resistant (level 0; L0). Representative phenotypes of highly PMD susceptible (L5), medium sensitivity (L3), and resistant (L0) are shown in **Figure 1A**, and the PMD resistance (L0) or susceptible level (L1–L5) of each accession is detailed in **Supplementary Table 1**. During the spring of 2018, we identified 196 resistant and 97 susceptible lines at the Hainan Experimental Station (**Figure 1B**). During the spring of 2019, we identified 207 resistant and susceptible 98 lines at the experimental station at South China Agricultural University (SCAU) (**Figure 1B**). During the spring of 2020, we identified 205 resistant and 110 susceptible accessions at the SCAU experimental station(**Figure 1B**). Pooling these results across years and locations, we found that 206 (66.35%) of the 315 accessions are resistant to PMD, and 109 (34.60%) are susceptible. These results indicate that soybean accessions are a rich resource for PMD resistance (**Supplementary Table 1**).

### 3.2 SNPs/InDels genotyping and GWAS for PMD resistance

We performed whole-genome re-sequencing of 315 soybean accessions from China, the United States, Brazil, Australia, and Africa (**Supplementary Table 2**), with an average 15× depth of sequencing. We mapped 92.65% of the reads to the soybean reference genome (Wm82.a4.v1 version) and used GATK to call 4,900,642 high-quality SNPs and 702,316 InDels (<40 bp) at bi-allelic loci. A distribution analysis of SNPs and InDels across the 20 chromosomes of the soybean genomes shows a high density near the ends of chromosomes 3, 6, 15, 16, and 18 (**Supplementary Figure 1**). Each chromosome has an average of 245,032 SNPs and 35,116 InDels.

We used the SNPs and InDels from the diversity collection of 315 accessions to conduct a GWAS with a mixed linear model approach to identify variant sites that are significantly associated with PMD resistance (**Figures 1C–F**). A total of 56 SNPs surpassed the genome-wide significance threshold clustered at the end of the long arm of chromosome 16 (**Figure 1G**). The SNP locus with the highest −log10 (p-value) of 19.14 is at nucleotide position 37,418,263 on Chr16 and accounts for 33.38% (R2) of the phenotypic variance. This GWAS peak is flanked by two paralogous R genes (*Glyma.16G214200* and *Glyma.16G214300*). Two other genes (*Glyma.16G213700* and *Glyma.16G214641*) overlap several SNPs in peaks with the second highest significance (**Figure 1G**). We defined 13 genes in this region as candidate PMD resistance genes (**Supplementary Table 3**). Furthermore, this region encompasses 8 NLR genes potentially associated with resistance to PMD, thereby yielding a total of 21 candidate genes for subsequent comprehensive analysis (**Supplementary Table 3**).

We used LD Blockshow to detect haplotype blocks in the 400-kb region containing the most significant SNP markers (**Figure 1G**). There is strong linkage disequilibrium across the region. The most significant SNP out of 60 significant SNPs in the block (located at Chr16: 37,416,562–37,418,263) (**Supplementary Figure 2**) is upstream of *Glyma.16G214200* and downstream of *Glyma.16G214300* suggesting that PMD resistance may be associated with variants in DNA regulatory elements of *Glyma.16g214200* or *Glyma.16g214300*.

### 3.3 Haplotype analysis of the candidate genes

**Figure 2:**
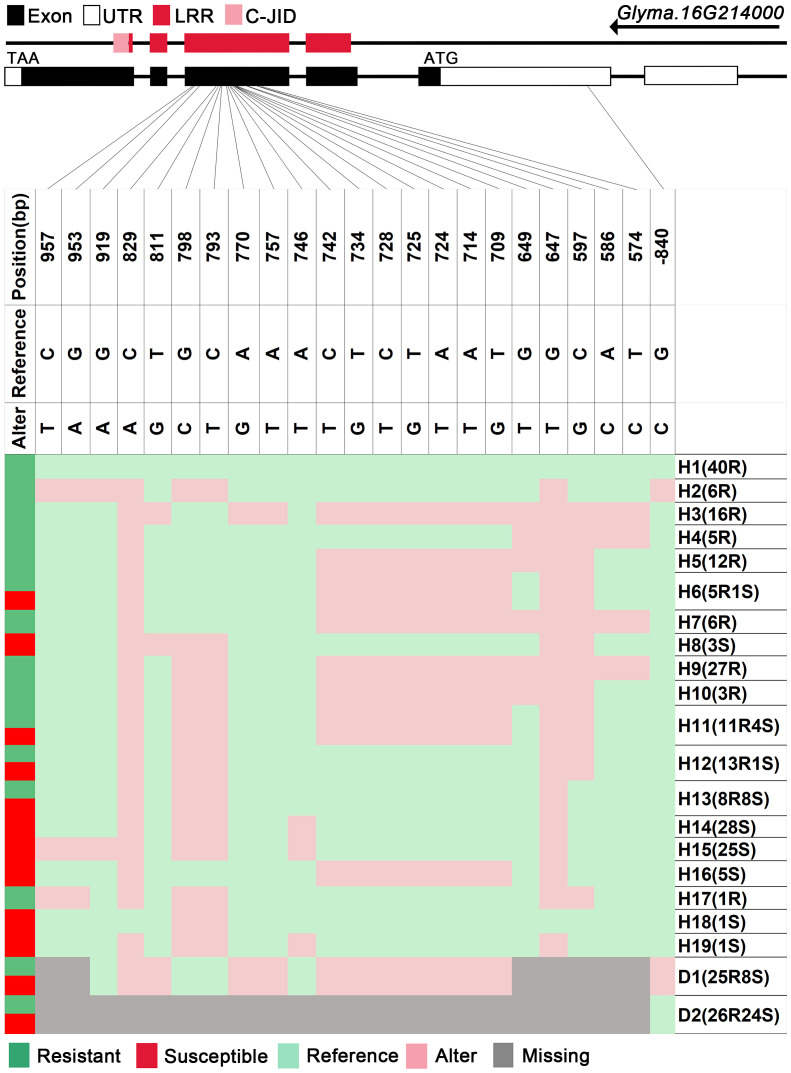
Haplotype analysis of *Glyma.16G214000*. H, haplotype; R, resistant; S, susceptible; D1, deletion type 1; H6(5R1S), five accessions in H6 are resistant, and one is susceptible; LRR, leucine-rich repeat domin; C-JID, C-terminal jelly roll/Ig-like domain.

**Figure 3:**
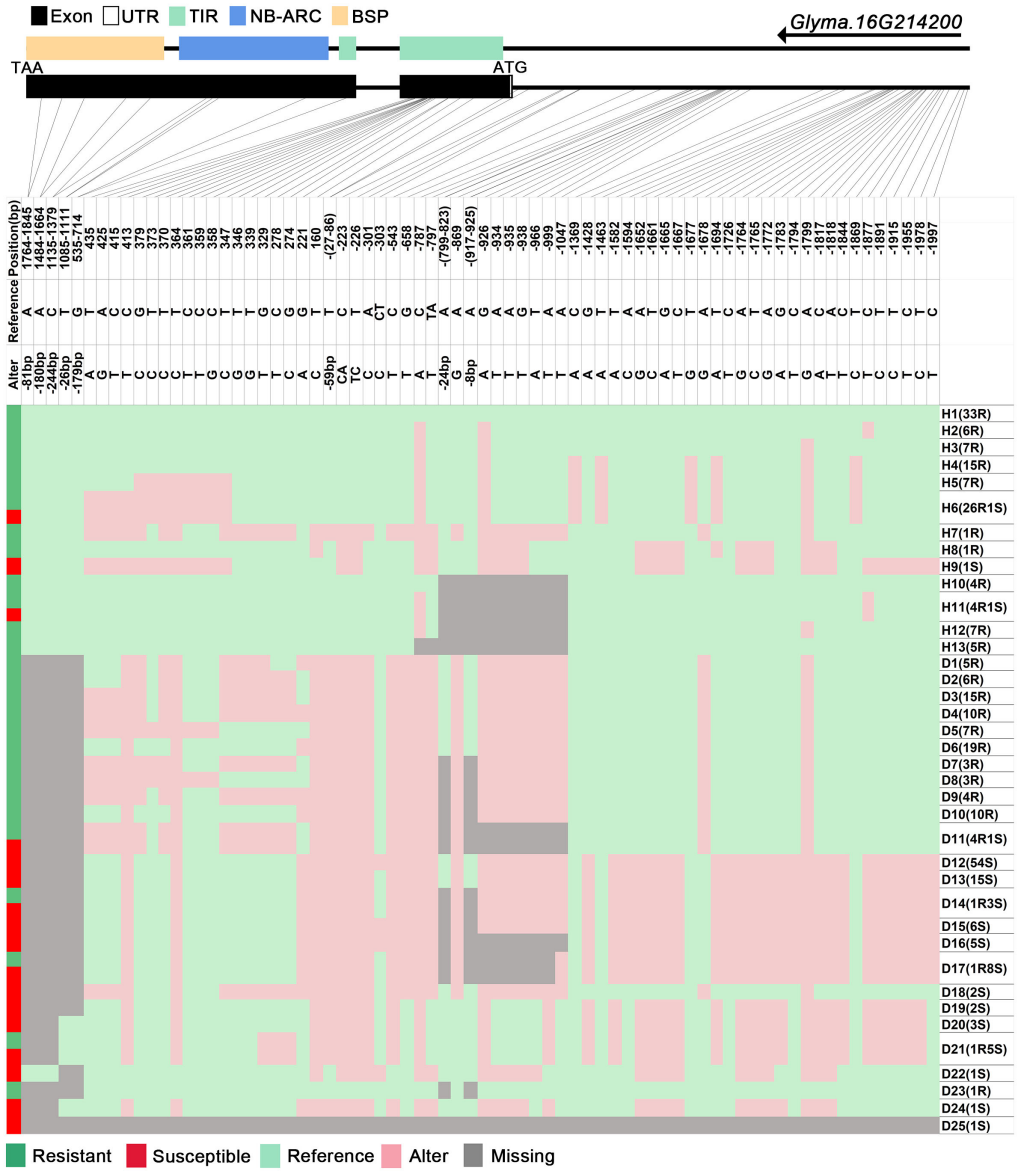
Haplotype analysis of *Glyma.16G214200*. H, haplotype; R, resistant; S, susceptible; D1, deletion type 1; H6(26R1S), 26 accessions in H6 are resistant, and 1 is susceptible; TIR, Toll/interleukin-1 receptor; NBS, nucleotide-binding site; BSP, basic secretory proteins.

**Figure 4:**
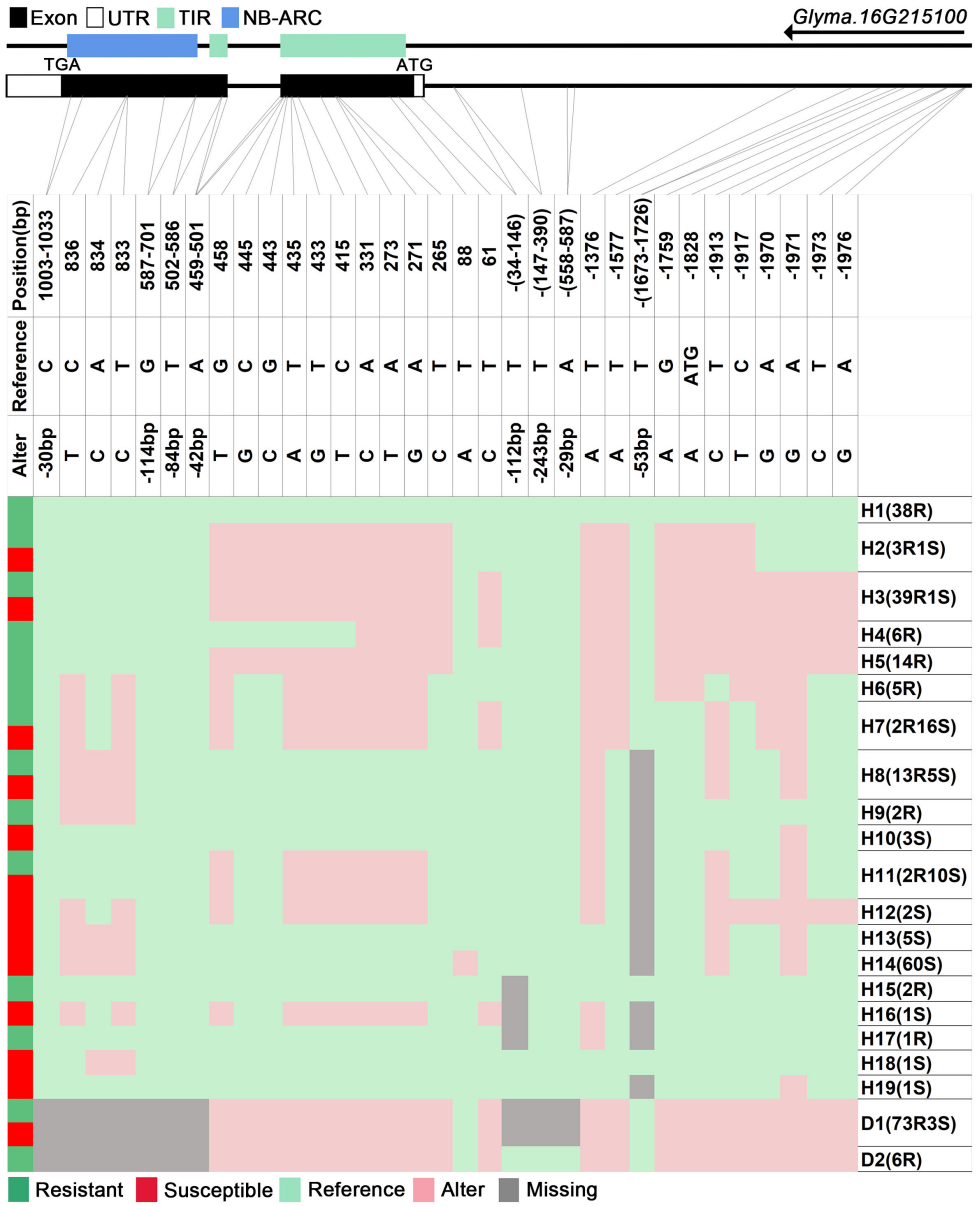
Haplotype analysis of *Glyma.16G215100*. H, haplotype; R, resistant; S, susceptible; D1, deletion type 1; H2(3R1S), three accessions in H2 are resistant, and one is susceptible; TIR, Toll/interleukin-1 receptor; NBS, nucleotide-binding site.

**Figure 5:**
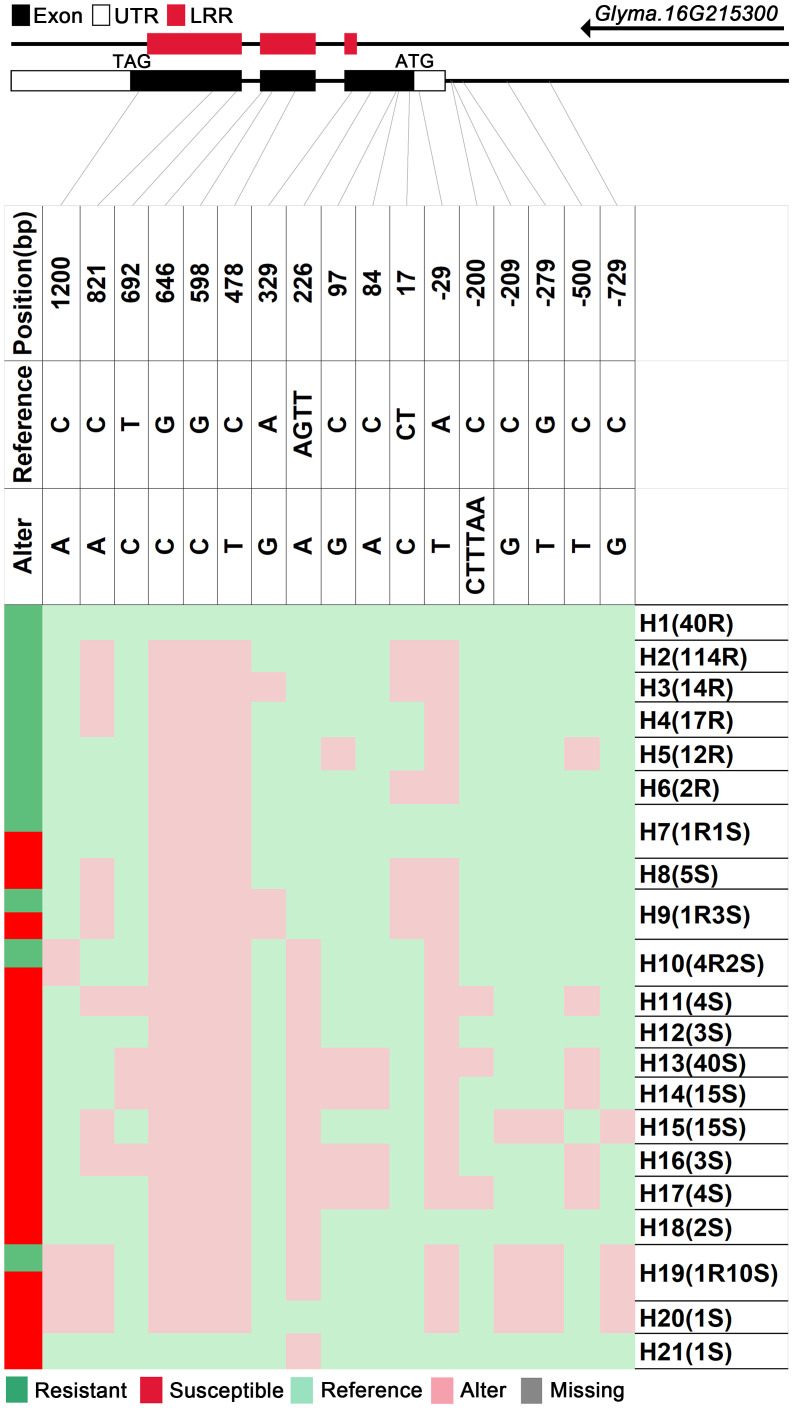
Haplotype analysis of *Glyma.16G215300*. H, haplotype; R, resistant; S, susceptible; D1, deletion type 1; H7(1R1S), one accession in H7 is resistant, and one is susceptible; LRR, leucine-rich repeat domin.

We analyzed SNPs and InDels at the 21-gene (*Glyma.16G213700*–*Glyma.16G215400*) locus in the diversity pane of 315 soybean accessions. Unsupervised clustering was used to cluster all SNP variant types based on three genotype codes: reference homozygote, SNP/InDel mutation (Alter), and missing (**Figure 2**). There is a mutation caused by a base substitution at position 746 bp in the coding region of the *Glyma.16G214000*, which is associated with 71.56% (78/109, out of 109 susceptible accessions, 78 contained the mutation) of susceptible accessions (**Figure 2**). SNP mutations in the upstream promoter region of *Glyma.16G214200* from −1,534 to −1,978 bp are associated with 95% (104/109) of susceptible accessions (**Figure 3**). Furthermore, several NLR candidate genes with relatively high peak values are located between *Glyma.16G214641* and *Glyma.16G215400* suggesting their potential involvement in PMD resistance. Haplotype analysis of these genes identified an SNP at position 72 in the coding region of the *Glyma.16G215100* gene, and this mutation was present in 55% (60/109) of susceptible accessions (**Figure 4**). There is a 3-bp deletion at 226 bp in the coding region of *Glyma.16G215300*, and 91% (99/109) of susceptible accessions are associated with this mutation (**Figure 5**). The haplotypes of other candidate genes are depicted in **Supplementary Figures 3–17**. In summary, SNP/InDel mutations in the coding and promoter regions of the above genes are closely related to their disease resistance, and these genes may be candidate genes for resistance to PMD.

### 3.4 Haplotype analysis of other candidate genes in 11 special accessions at the *GmRmd1* locus

**Figure 6:**
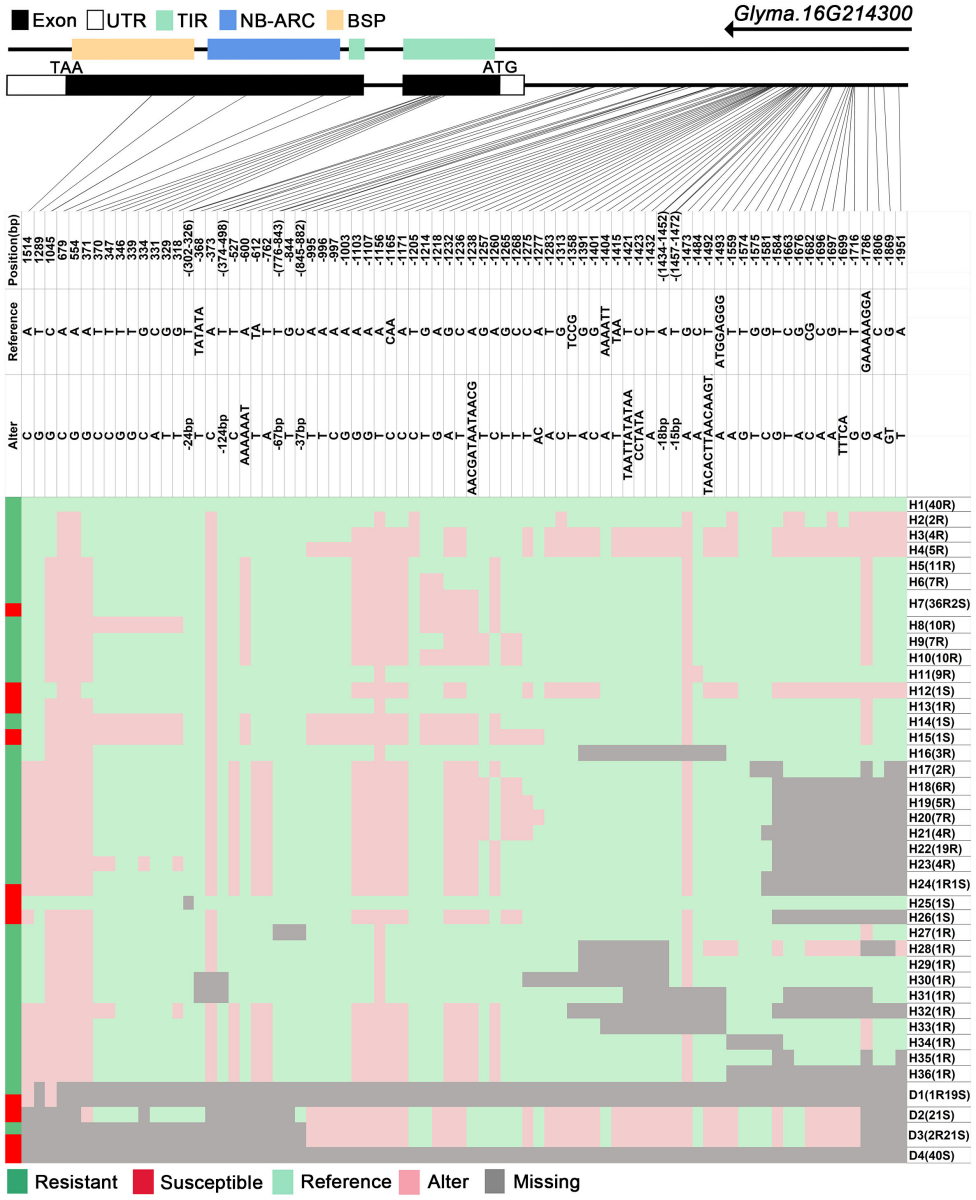
**|.** Haplotype analysis of *Glyma.16G214300*. H, haplotype; R, resistant; S, susceptible; D1, deletion type 1; H7(36R2S), 36 accessions in H6 are resistant, and 2 are susceptible; TIR, Toll/interleukin-1 receptor; NBS, nucleotide-binding site; BSP, basic secretory proteins.

The *GmRmd1* (*Glyma.16G214300*) gene has been previously confirmed as a resistant gene against PMD in various studies (Xian et al., 2022). In this study, the majority of accessions possessing intact *GmRmd1* coding regions demonstrate PMD resistance, whereas those lacking such regions exhibit susceptibility to PMD (**Figure 6**). These findings underscore the critical importance of maintaining an intact coding region for *GmRmd1* in conferring soybean resistance against accessions. However, the integrity of the *GmRmd1* coding region was not consistent with the PMD phenotype in 11 of the 315 accessions in which three of the resistant accessions exhibited incomplete *GmRmd1*; concurrently, eight of the susceptible accessions displayed intact *GmRmd1* (**Supplementary Table 4**). This suggests the presence of additional genetic factors contributing to resistance against PMD. Subsequently, through the analysis of haplotypes in other candidate genes (*Glyma.16G213700*–*Glyma.16G215400*) within these 11 accessions, and by integrating high-quality haplotype data (**Supplementary Tables 4**, **5**), it was observed that among the eight susceptible accessions, haplotypes (D3 of *Glyma.16G213700*), (H18 and H19 of *Glyma.16G213800*), (H15 and H16 of *Glyma.16G214000*), (D18 of *Glyma.16G214200*), (H11 of *Glyma.16G214500*), (H8, H19, and D7 of *Glyma.16G214529*), (H9 of *Glyma.16G214557*), (H41 of *Glyma.16G214585*), (H37, H40, and D1 of *Glyma.16G214613*), (H32, H50, H51, and D1 of *Glyma.16G214641*), (H12 and H18 of *Glyma.16G215100*), and (H8 of *Glyma.16G215300*) were found to be susceptible in other accessions. Among the three disease-resistant accessions, haplotypes (H4 of *Glyma.16G214529*), (H29 and H36 of *Glyma.16G214585*), (H8 of *Glyma.16G214613*), (H6 of *Glyma.16G215100*), and (H2 of *Glyma.16G215300*) exhibited resistance to diseases in other accessions. In conclusion, it is plausible that *Glyma.16G213700*, *Glyma.16G213800*, *Glyma.16G214000*, *Glyma.16G214200*, *Glyma.16G214500*, *Glyma.16G214557*, *Glyma.16G214585*, *Glyma.16G214613*, *Glyma.16G214641*, *Glyma.16G215100*, and *Glyma.16G215300* may represent additional genes exhibiting resistance against PMD.

### 3.5 The assessment of the integrity of coding regions in candidate genes

**Figure 7:**
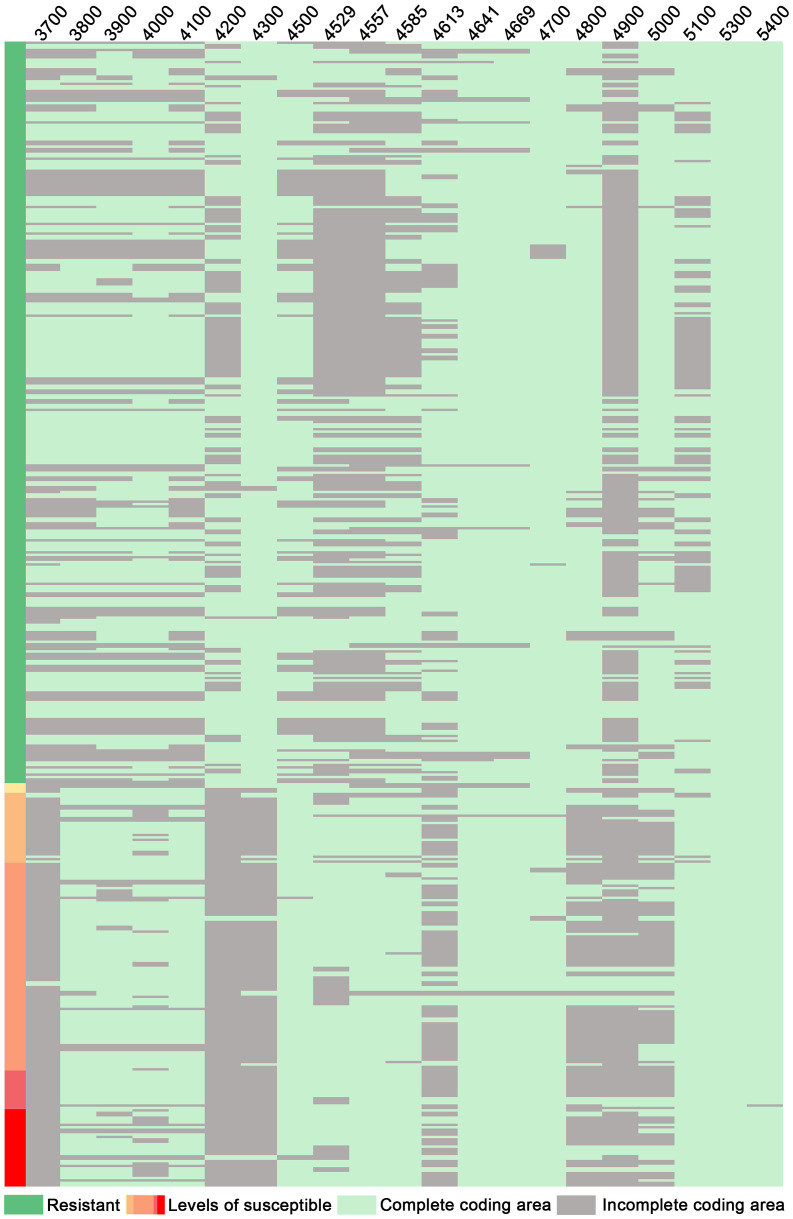
Integrity analysis of candidate gene-coding regions: 3700: *Glyma.16g213700*; 3800: *Glyma.16g213800*, and so on. Levels of susceptibility: Disease levels 1 to 5.

Through structural analysis of the coding regions of candidate genes, it was observed that among 206 disease-resistant accessions, a total of 203 exhibited intact *Glyma.16G214300*; conversely, out of 109 susceptible accessions, only 101 displayed incomplete *Glyma.16G214300* (**Figure 7**). Furthermore, within the cohort of disease-resistant accessions, a significant majority (193 out of 206) possessed intact *Glyma.16G214800*; in contrast, among the susceptible group (consisting of 109 samples), only 82 showed incomplete *Glyma.16G214800* (**Figure 7**). Similarly, within the population of disease-resistant accessions (206 in total), a substantial proportion (185) demonstrated intact *Glyma.16G215000*; however, among the susceptible counterparts (comprising 109 samples), merely 68 exhibited incomplete *Glyma.16G215000* (**Figure 7**). This observation suggests a strong association between the integrity of the coding region for *Glyma.16G214300*, *Glyma.16G214800*, and *Glyma.16G215000* and their disease resistance phenotypes, and the resistance gene *Glyma.16G214300* has been confirmed against PMD (Xian et al., 2022) suggesting that genes *Glyma.16G214800* and *Glyma.16G215000* are highly likely to confer resistance to PMD as well. It is plausible that these genes form a complex network involved in conferring disease resistance by synergistically exerting protective effects against PMD.

### 3.6 Expression patterns for candidate genes

**Figure 8:**
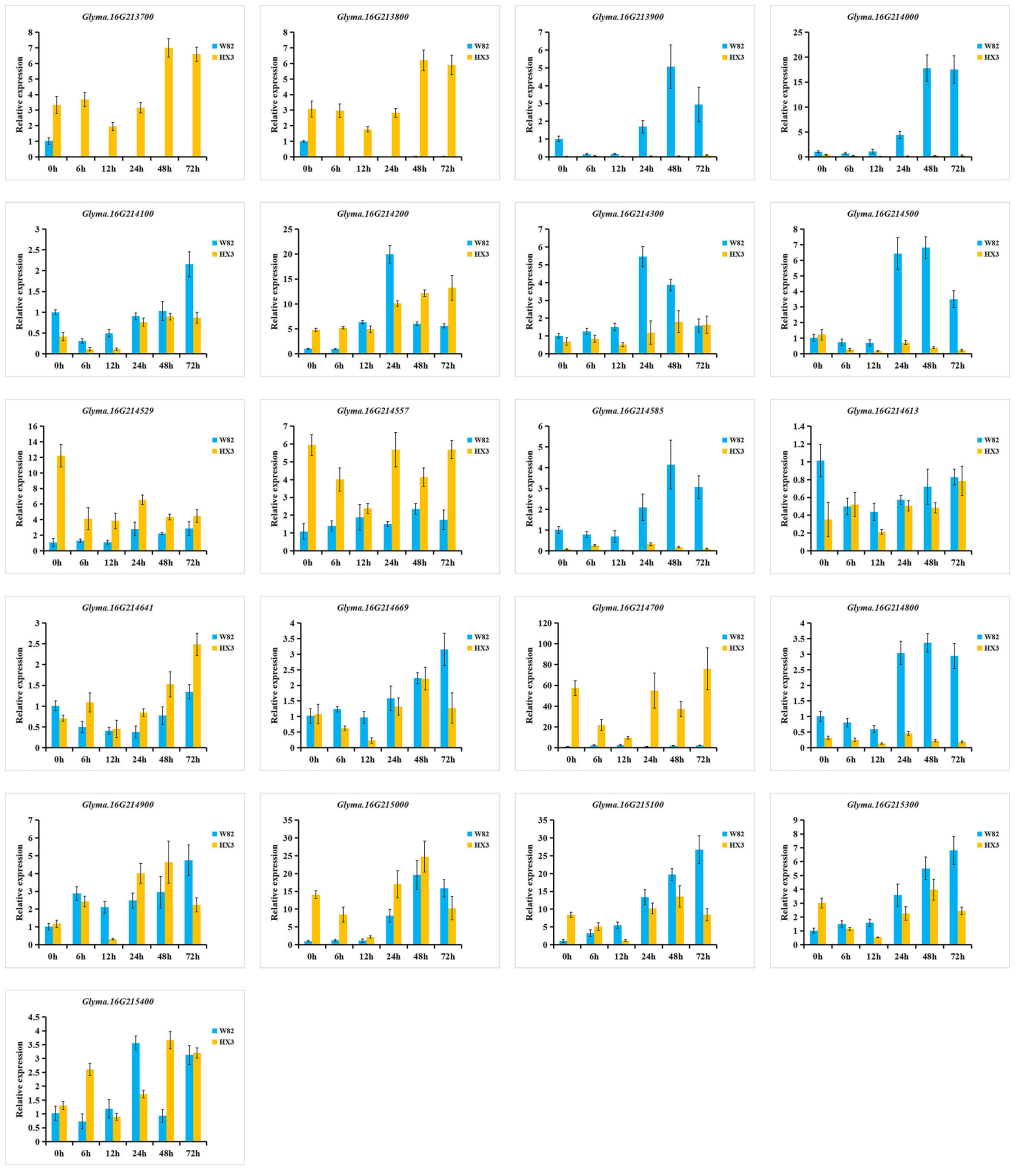
Relative expression levels of 21 candidate genes in W82 (resistant) and HX3 (susceptible). qRT-PCR analysis of the expression of 21 candidate genes at 0, 6, 12, 24, 48, and 72 h after an *M. diffusa* spore suspension containing 1 × 10^5^ cfu/ml treatment. The data are shown as mean ± SD.

To investigate the expression of 21 candidate genes in response to *M. diffusa* infection, qRT-PCR was utilized to examine the expression patterns of each gene between Williams 82 (W82, resistance to PMD) and Huaxia 3 (HX3, susceptible to PMD) (**Figure 8**). Among these genes, the expression levels of *Glyma.16G213900* and *Glyma.16G214000* were predominantly observed in W82, while they were scarcely detected in HX3. In comparison to HX3, the expression levels of *Glyma.16G214100*, *Glyma.16G214300*, *Glyma.16G214500*, *Glyma.16G214585*, *Glyma.16G214800*, *Glyma.16G215100*, and *Glyma.16G215300* were upregulated highly in W82. Conversely, the expression levels of *Glyma.16G214529*, *Glyma.16G214557*, *Glyma.16G214641*, and *Glyma.16G215000* showed lower expression levels in W82 compared with HX3. The expression levels of *Glyma.16G214613* and *Glyma.16G214669* exhibited similar patterns between W82 and HX3. However, *Glyma.16G213700*, *Glyma.16G213800*, and *Glyma.16G214700* were expressed in HX3 but not detected in W82 suggesting their potential involvement in negative regulation of PMD resistance.

Additionally, six genes (*Glyma.16G213900*, *Glyma.16G214000*, *Glyma.16G214500*, *Glyma.16G214300*, *Glyma.16G214585*, and *Glyma.16G214800*) were induced by *M. diffusa* in W82 but showed minimal or low expression in HX3, and the expression levels of *Glyma.16G213900*, *Glyma.16G214000*, and *Glyma.16G214500* gradually increased in W82 after *M. diffusa* infection, reaching their peak after 48 h and subsequently decreasing. Correspondingly, the expression levels of the *Glyma.16G214300*, *Glyma.16G214585*, and *Glyma.16G214800* genes peaked after 24 h followed by a gradual decline. These results indicated that the above six genes could positively regulate PMD resistance. In addition, five genes (*Glyma.16G214200*, *Glyma.16G214669*, *Glyma.16G215000*, *Glyma.16G215100*, and *Glyma.16G215300*) were induced to express in W82 after being infected by *M. diffusa*. Among them, the expression level of *Glyma.16G214200* reached its peak at 24 h, while that of *Glyma.16G215000* peaked at 48 h. Similarly, the highest expression levels for *Glyma.16G214669*, *Glyma.16G215100*, and *Glyma.16G215300* were observed after 72 h. However, these five genes exhibited distinct expression patterns in HX3 at each time point, suggesting a potential coordinated role of these genes in the regulation of PMD, necessitating their collaboration with other resistance genes for effective control of *M. diffusa* infection. To summarize, a total of 14 genes may be involved in the regulation of PMD resistance, with three genes specifically implicated in negative regulation.

### 3.7 Williams 82 mutant lines confirm that multiple genes are involved in soybean PMD resistance

**Figure 9:**
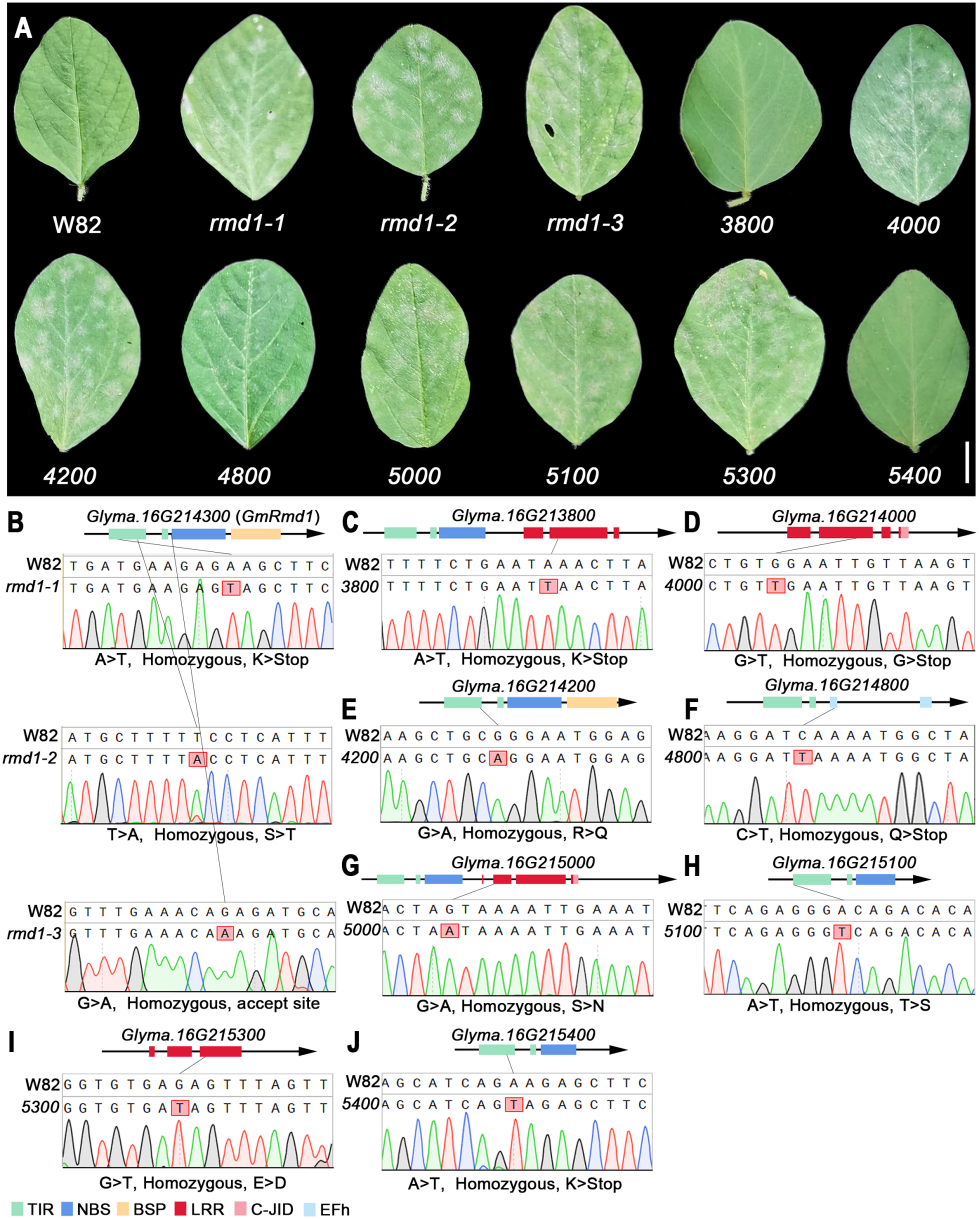
PMD resistance evaluation of candidate gene mutant lines. **(A)** Mutant phenotype of W82. W82: Williams 82, wild-type; *rmd1-1*, *rmd1-2*, *rmd1-3*: different mutants of *Glyma.16G214300*; *4000*, mutants of *Glyma.16G214000*, an so on. Scar bar: 1 cm. **(B–J)** Sequencing analysis of W82 mutant. **(B)** A > T/homozygous/K > Stop: Base A mutates into T/homozygous mutant/amino acid K mutates into codon terminator. **(C–J)** Consistent with the analysis method in **(B)**. TIR, Toll/interleukin-1 receptor; NBS, nucleotide-binding site; BSP, basic secretory proteins; LRR, leucine-rich repeat domin; C-JID, C-terminal jelly roll/Ig-like domain; EFh, EF-hand domain.

Mutant lines ranging from *3700* (*Glyma.16G213700*) to *5400* (*Glyma.16G215400*), derived from Williams 82 induced with EMS, were obtained from Song’s laboratory (Zhang et al., 2022), and the specific mutation sites and homozygosity of each line are depicted in **Figures 9B–J** and **Supplementary Table 7**. Based on the phenotypic characteristics of mutant lines, it can be inferred that *rmd1-1*, *rmd1-2*, *rmd1-3*, *4000*, *4200*, *4800*, *5000*, *5100*, and *5300* lines exhibit susceptibility to PMD, while the mutant lines of other candidate genes, such as *3800* and *5400*, are resistant to PMD (**Figure 9A**). Among them, the gene *Glyma.16G214300* in the mutant lines *rmd-1*, *rmd-2*, and *rmd-3* has been identified as a known resistance gene against PMD (Xian et al., 2022). However, *Glyma.16G214300* was not mutated in lines *3800*, *4000*, *4200*, *4800*, *5000*, *5100*, *5300*, and *5800* (**Supplementary Figures 18**-**22**). This suggests that apart from *Glyma.16G214300*, *Glyma.16G214000*, *Glyma.16G214200*, *Glyma.16G214800*, *Glyma.16G215000*, *Glyma.16G215100*, and *Glyma.16G215300* also significantly contribute to PMD resistance.

## 4 Discussion

PMD, caused by the fungus *M. diffusa*, has resulted in substantial yield losses for soybeans. The identification of resistant genes and the breeding of resistant cultivars under optimal environmental conditions can effectively mitigate the detrimental impact imposed by soybean PMD. Previous studies used populations to identify soybean PMD resistance loci, which are concentrated at the end of Chr16 (35,968,531–37,733,538 bp; use Wm82.a2.v1 reference genome). These loci are *Rmd_V97-3000* at 34,035,391–37,631,694 (Wang et al., 2013), *Rmd_PI24540* at 34,258,523–36,750,257 (Kang and Mian, 2010), *PMD_PI567301B* (5000–6700) at 37,249,583–37,370,175 bp (Jun et al., 2012), *Rmd_B3* at 36,221,397–37,631,694 (Jiang et al., 2019), *Rmd_B13* at 37,102,014-37,290,074 (Jiang et al., 2019), *Rmd_ZH24* at 37,202,495–37,235,283 (Zhou et al., 2022), and *Rmd_ZDD00359* at 37,011,583–37,234,234 (Sang et al., 2023). They completely or partially overlap one another and may be an R-gene cluster or a single locus. In these loci, *Rmd_V97-3000*, *Rmd_B3*, *Rmd_B13*, *Rmd_ZH24*, and *Rmd_ZDD00359* all harbor the *GmRmd1* gene. Additionally, it was later confirmed that the resistance gene in *Rmd_B13* is indeed *GmRmd1*. Besides these findings, the loci *Rmd_PI24540* and *PMD_PI567301B* do not possess the *GmRmd1* gene indicating the presence of other yet-to-be-identified genes responsible for PMD resistance. The *PMD_PI567301B* locus harbors 16 genes, including *Glyma.16G215000*, *Glyma.16G215100*, *Glyma.16G215300*, and *Glyma.16g215400* as NLR genes, suggesting that one of these four genes may confer resistance to soybean PMD caused by *PMD_PI567301B*. Hu (2021) analyzed 331 soybean accessions through GWAS and identified three candidate genes (*Glyma.16g210800*, *Glyma.16g211000*, and *Glyma.16g211400*) treated as PMD resistance genes. Another study also identified three candidate genes (*Glyma.16G210800*, *Glyma.16G212300*, and *Glyma.16G213900*) that were considered PMD-resistant genes by GWAS analysis (Sang et al., 2023).The results indicate that different accessions resistant to PMD may carry various resistance genes.

In this study, we used GWAS to identify a region on the end of Chr16 associated with PMD resistance (**Figure 1E**). This region contains 21 genes, with the exception of *Glyma.16G214613* and *Glyma.16G214641*, as the remaining 19 genes all belong to the NLR gene family (**Supplementary Figure 23**). Among these, the gene *Glyma.16G214200* encodes a protein that demonstrates similarity to *GmRmd1* with a BSP (basic secretory protein, peptidase of plants and bacteria) domain. The BSP domain has similarity to the defense-related proteins *WCI-5* from wheat (Görlach et al., 1996), *NtPRp27* from tobacco (Okushima et al., 2000), and *StPRp27* from potato (Shi et al., 2012). *GmRmd1* and *Glyma.16G214200* are homologs of *WCI-5/NtPRp27*, with a shared homology of 84% (**Supplementary Figure 24**), indicating that *Glyma.16G214200* may play an important role in resistance to PMD. Recently, experimental evidence has demonstrated that the truncated protein TIR-NBS of *SRC7* (*GmRmd1*) exhibits robust resistance against SMV and TMV (Yan et al., 2022). Moreover, partial resistance is also observed in the truncated protein TIR-NBS of *SRC8* (*Glyma.16G214200*) suggesting that the BSP domain is not dispensable for antiviral activity (Yan et al., 2022). Through structural domain and evolutionary analysis of candidate proteins (**Supplementary Figures 23**, **24**), it was discovered that *Glyma.16G215100* solely contains the TIR and NBS domains without the LRR or BSP domains at its C-terminus. Additionally, *Glyma.16G215100* shares significant similarity with truncated protein TN from *Glyma.16G214200* and *Glyma.16G214300* displaying strong resistance to both soybean mosaic virus (SMV) and tobacco mosaic virus (TMV) (Yan et al., 2022). These findings imply a potentially crucial role of *Glyma.16G215100* in PMD resistance.

Calcium ion-related compounds also play a crucial role in plant immune defense with many calcium-binding proteins containing EF-hand-type calcium-binding domains (Ikura et al., 2002; Nelson et al., 2002). The SRC4 (Glyma.16G214800) proteins possess a Toll/interleukin-1 receptor (TIR) domain at the N-terminus and an EFh (EF-hand) domain at the C-terminus (**Supplementary Figure 23**), and proteins harboring these domains are implicated in conferring resistance against SMV and TMV (Yan et al., 2022) suggesting that *Glyma.16G214800* may also contribute to PMD resistance. The genes *Glyma.16G214000* and *Glyma.16g215000* are all NLR family genes that possess the C-JID domain (**Supplementary Figure 23**), and this domain is responsible for substrate recognition by binding to effector proteins of pathogens and plays a crucial role in initiating TIR-NLR receptor signaling. The RPS4 protein in Arabidopsis, which contains the C-JID (or posterior LRR) structure, collaborates with RRS1 to recognize effectors and subsequently exert disease resistance (Ma et al., 2020). The C-terminus of *GmRmd1* harbors a BSP domain while lacking the LRR domain responsible for direct pathogen recognition. Proteins possessing the C-JID domain, such as *Glyma.16G214000* and *Glyma.16g215000*, may interact with *GmRmd1* to collectively perceive effector factors and confer disease resistance effects.

Examining differential gene expression patterns has been regarded as a promising approach for gaining a deeper biological understanding of GWAS signals (Emilsson et al., 2008). In this study, we identified 21 candidate genes associated with PMD resistance through GWAS and haplotype analysis. Among them, three genes (*Glyma.16G213700*, *Glyma.16G213800*, and *Glyma.16G214700*), which are expressed only in HX3 but not in W82, may potentially compete with resistance genes for binding to other resistance genes during the regulation of PMD resistance, thereby exerting a negative regulatory effect on PMD resistance. In addition, 11 genes (*Glyma.16G213900*, *Glyma.16G214000*, *Glyma.16G214200*, *Glyma.16G214300*, *Glyma.16G214500*, *Glyma.16G214585*, *Glyma.16G214669*, *Glyma.16G214800*, *Glyma.16G215000*, *Glyma.16G215100*, and *Glyma.16G215300*) were induced by *M. diffusa* in W82, while 6 genes (*Glyma.16G213900*, *Glyma.16G214000*, *Glyma.16G214500*, *Glyma.16G214300*, *Glyma.16G214585*, and *Glyma.16G214800*) exhibited either repressed or constrained levels of expression in HX3 indicating that these 6 genes play important roles in PMD. However, the other five genes (*Glyma.16G214200*, *Glyma.16G214669*, *Glyma.16G215000*, *Glyma.16G215100*, and *Glyma.16G215300*) showed different expression patterns at different time points in HX3 indicating that these five genes may need to work together with other genes to act on *M. diffusa*. Previous studies have demonstrated that genes exhibiting differential expression patterns between accessions are frequently directly or indirectly associated with susceptibility or resistance outcomes. Conversely, genes displaying distinct expression dynamics over time may represent a general plant response to pathogen infection without necessarily conferring increased resistance (Calla et al., 2009). Therefore, the aforementioned 11 genes can be regarded as robust candidate genes for conferring resistance against PMD. Combined with haplotype, qRT-PCR, and mutant analysis data, it was ultimately determined that seven genes (*Glyma.16G214000*, *Glyma.16G214200*, *Glyma.16G214300*, *Glyma.16G214800*, *Glyma.16G215000*, *Glyma.16G215100*, and *Glyma.16G215300*) were identified as being important in PMD defense.

In this study, numerous novel candidate genes for PMD resistance have been identified. However, their mode of action remains uncertain, whether they function independently or synergistically with the *GmRmd1*. It is plausible that these genes gradually enhance resistance to PMD through functional superposition. Drawing inspiration from the strategy employed in generating PMD-resistant and high-yielding *Tamlo R32* wheat mutants (Li et al., 2022), genome editing emerges as an appealing approach for progressively eliminating or introducing disease-resistant genes in both resistant and susceptible soybeans. In conclusion, this study identified several resistance genes except *GmRmd1* that was closely associated with PMD resistance, and these results provide important genetic resources for breeding scientists to develop PMD resistance accessions.

## 5 Conclusion

Several important pathogens, including PMD, occur in soybeans and cause significant yield reductions globally. In this study, a genome-wide association study (GWAS) identified SNPs and InDels significantly associated with PMD resistance in a cluster of disease-resistant genes located at the distal end of chromosome 16. Haplotype, qRT-PCR, and mutant analysis revealed that candidate genes for resistance against PMD include *Glyma.16g214000*, *Glyma.16g214200*, *Glyma.16g214300*, *Glyma.16g214800*, *Glyma.16g215000*, *Glyma.16g215100*, and *Glyma.16g215300*. These findings establish a robust genetic basis for further elucidating the mechanisms underlying PMD resistance and facilitating breeding efforts toward developing resistant accessions.

## Data availability statement

The datasets presented in this study can be found in online repositories. The names of the repository/repositories and accession number(s) can be found in the article/**Supplementary Material**.

## Author contributions

GL: Writing – review & editing, Investigation, Formal analysis, Data curation. YF: Writing – review & editing, Validation, Formal analysis. XL: Writing – review & editing, Investigation, Data curation. JJ: Writing – review & editing, Investigation, Data curation. GD: Writing – review & editing, Investigation. YZW: Writing – review & editing, Data curation. XZ: Writing – review & editing, Data curation. XX: Writing – review & editing, Data curation. ML: Writing – review & editing, Investigation, Data curation. YXW: Writing – review & editing, Writing – original draft, Supervision, Data curation. CY: Writing – review & editing, Writing – original draft, Supervision, Resources, Funding acquisition, Conceptualization.
