# Supplementary material for: Genome-wide association study and haplotype analysis reveal novel candidate genes for resistance to powdery mildew in soybean
Source: Front Plant Sci. 2024 Mar 27;15:1369650. doi: 10.3389/fpls.2024.1369650 (PMC11019568; doi:10.3389/fpls.2024.1369650)
Supplement: Supplementary file 1 [file DataSheet_1.zip › Data Sheet 3/Supplementary Figure 1-24.pdf]

Supplementary Material

Genome-wide association study and haplotype analysis reveal novel candidate genes for resistance to powdery mildew in soybean

Guoqiang Liu[1,2]†, Yuan Fang[3]†, Xueling Liu[1,2]†, Jiacan Jiang[1,2], Guangquan Ding[1,2], Yongzhen Wang[1,2], Xueqian Zhao[1,2], Xiaomin Xu[1,2], Mengshi Liu[1,2], Yingxiang Wang[3,4]*, Cunyi Yang[1,2]*

[1]Guangdong Provincial Key Laboratory of Plant Molecular Breeding, College of Agriculture, South China Agricultural University, Guangzhou, China, [2]Key Laboratory for Enhancing Resource Use Efficiency of Crops in South China, Ministry of Agriculture and Rural Affairs, South China Agricultural University, Guangzhou, China, [3]Guangdong Laboratory for Lingnan Modern Agriculture, Guangzhou, China, [4]Guangdong Provincial Key Laboratory of Protein Function and Regulation in Agricultural Organisms, College of Life Sciences, South China Agricultural University, Guangzhou, China

*** Correspondence:**

Cunyi Yang
ycy@scau.edu.cn

Yingxiang Wang

yx_wang@fudan.edu.cn

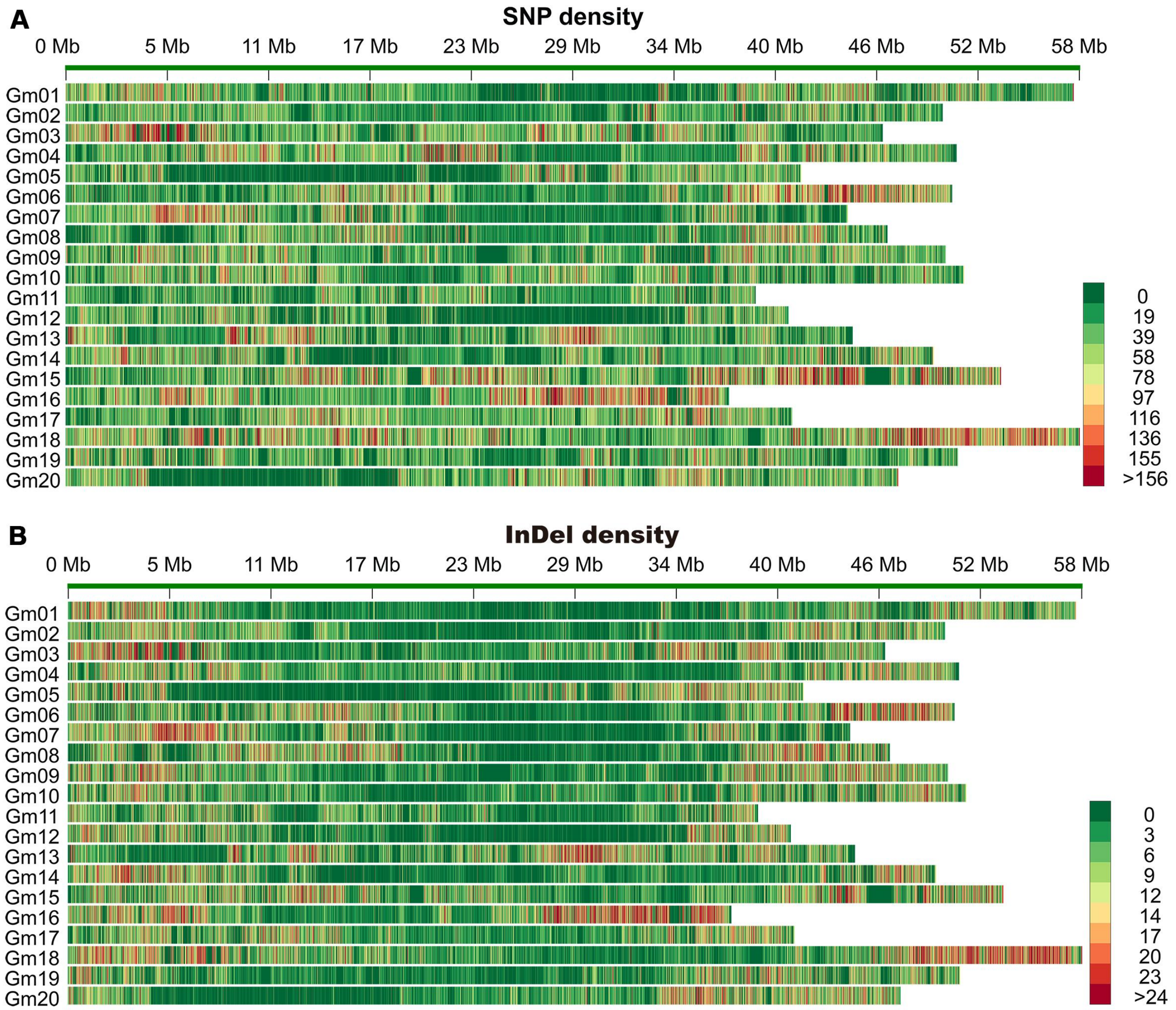

Supplementary Figure 1. The density of SNPs and InDels across all chromosomes in soybean. (A, B) Chromosomes were divided into 10 kb bins, and the number of SNPs (A) and InDels (B) in each bin is visualized using a heatmap. The colors from green to red represent the numbers of SNPs and InDels from low grade to high.

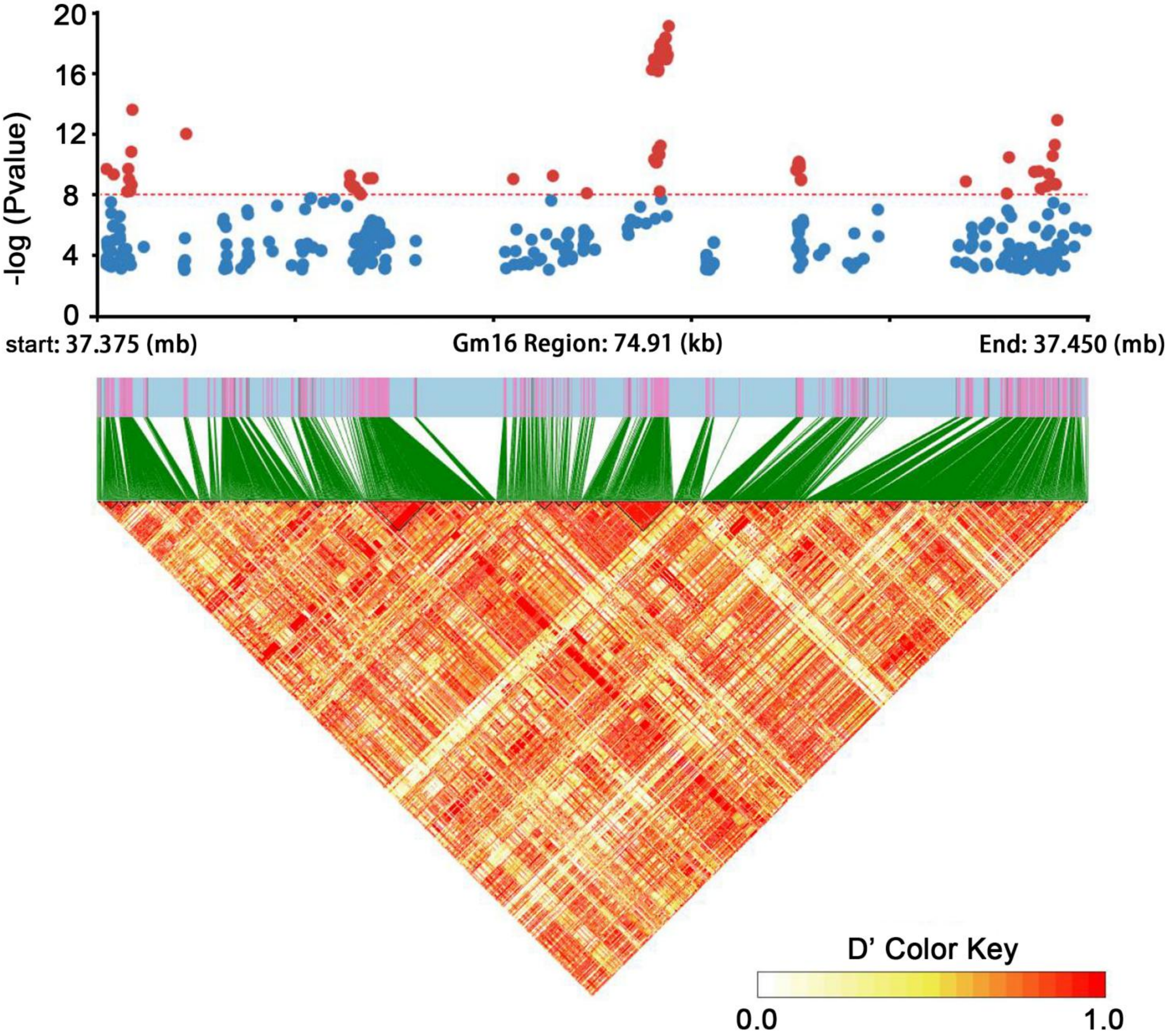

Supplementary Figure 2. Haplotype Blocks in the region of 13 clustered genes with significant GWAS SNPs on chromosome16. Local Manhattan plot and linkage disequilibrium heatmap of 13 clustered genes. Two distinct blocks were detected in this region, and 60 of most significant SNPs are present in one block which is located between *Glyma.16G214200* and *Glyma.16G214300*. Horizontal red dashed lines indicate the genome-wide significant threshold.

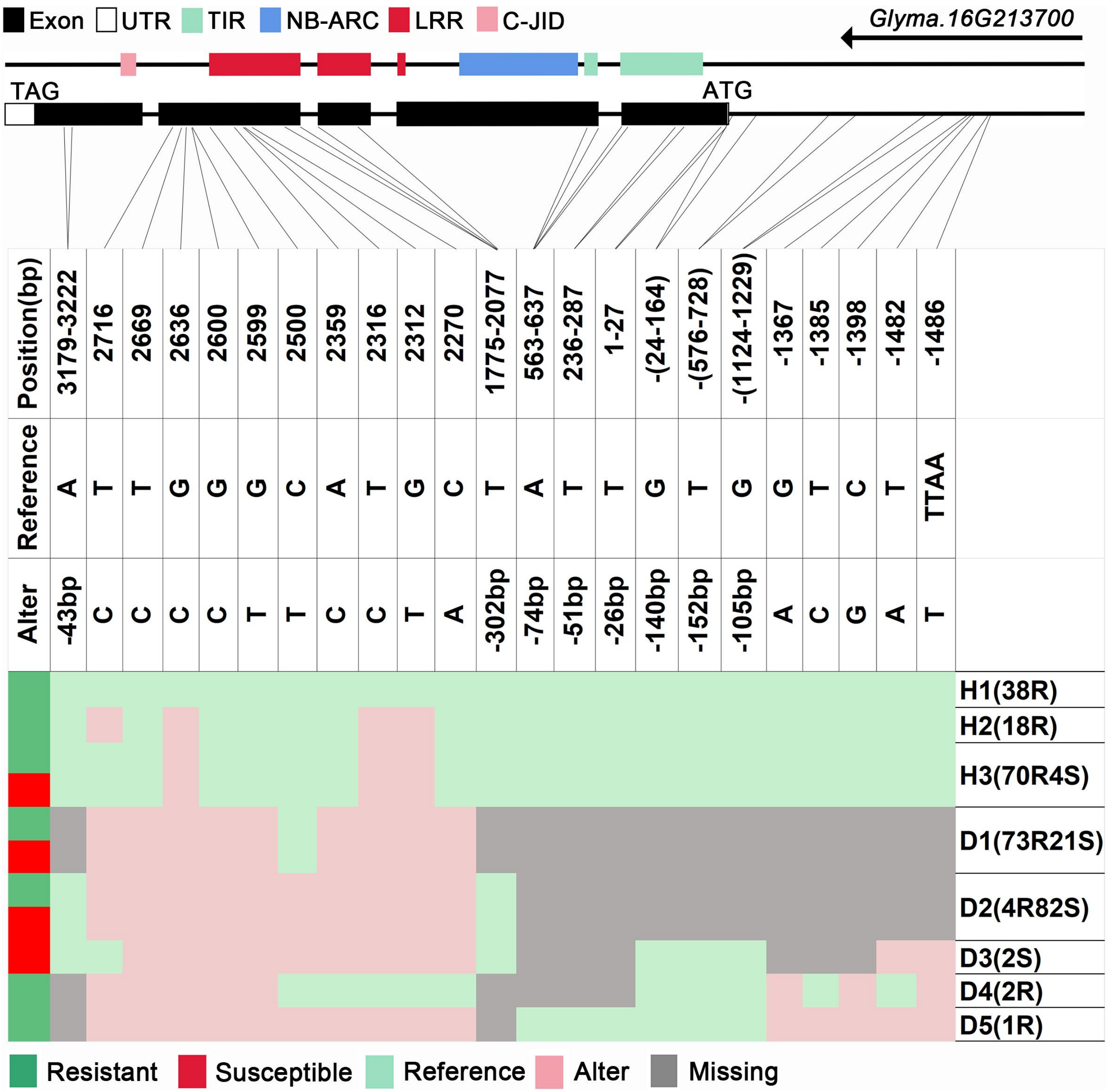

Supplementary Figure 3. Haplotype analysis of *Glyma.16g213700*. H:haplotype; R: Resistant; S: Susceptible; D1:Deletion type 1; TIR:Toll/interleukin-1 receptor; NBS:nucleotide-binding site. LRR:leucine-rich repeat domin; C-JID: C-terminal jelly roll/Ig-like domain.

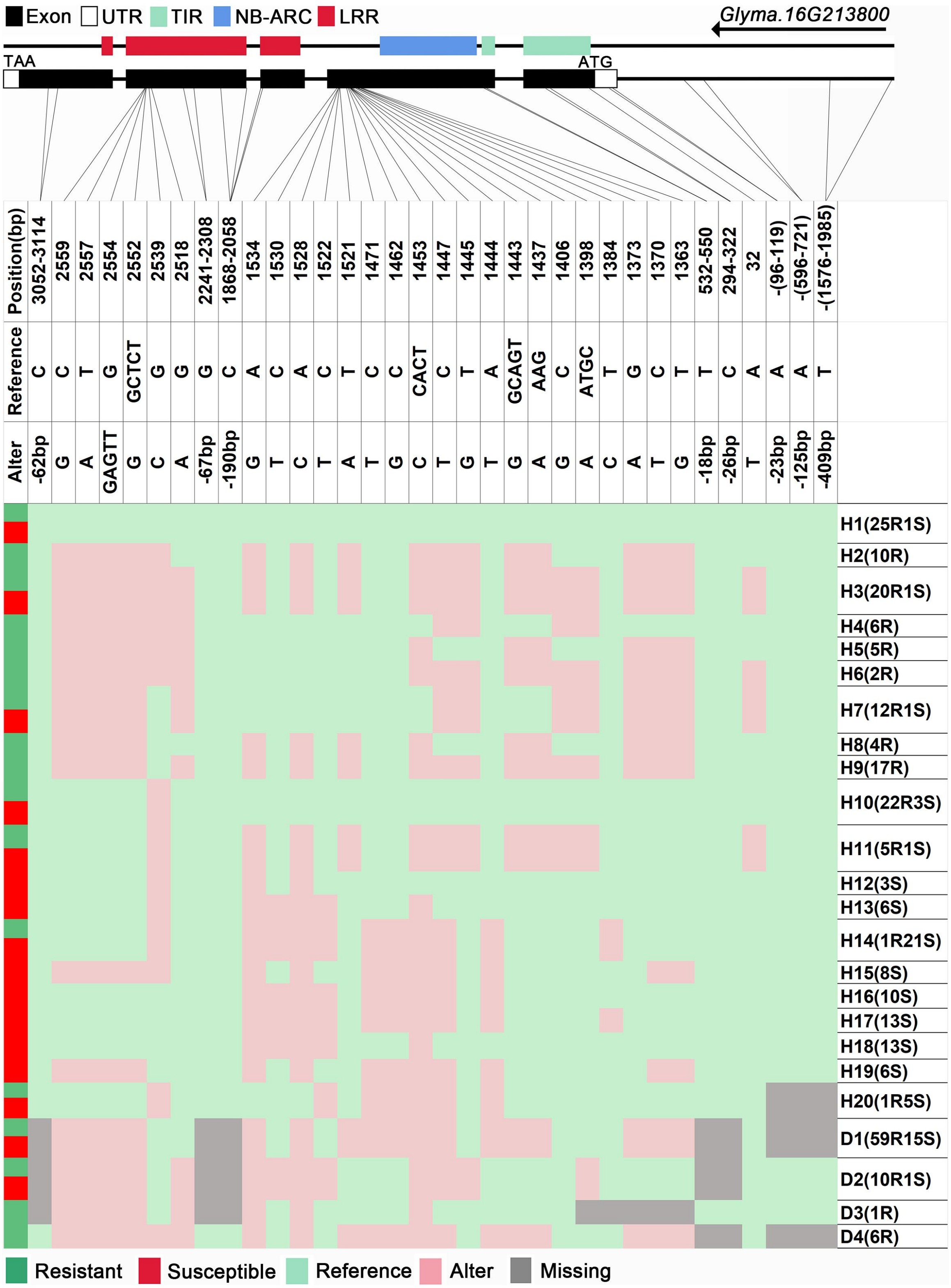

Supplementary Figure 4. Haplotype analysis of *Glyma.16g213800*. H:haplotype; R: Resistant; S: Susceptible; D1:Deletion type 1; TIR:Toll/interleukin-1 receptor; NBS:nucleotide-binding site. LRR:leucine-rich repeat domin.

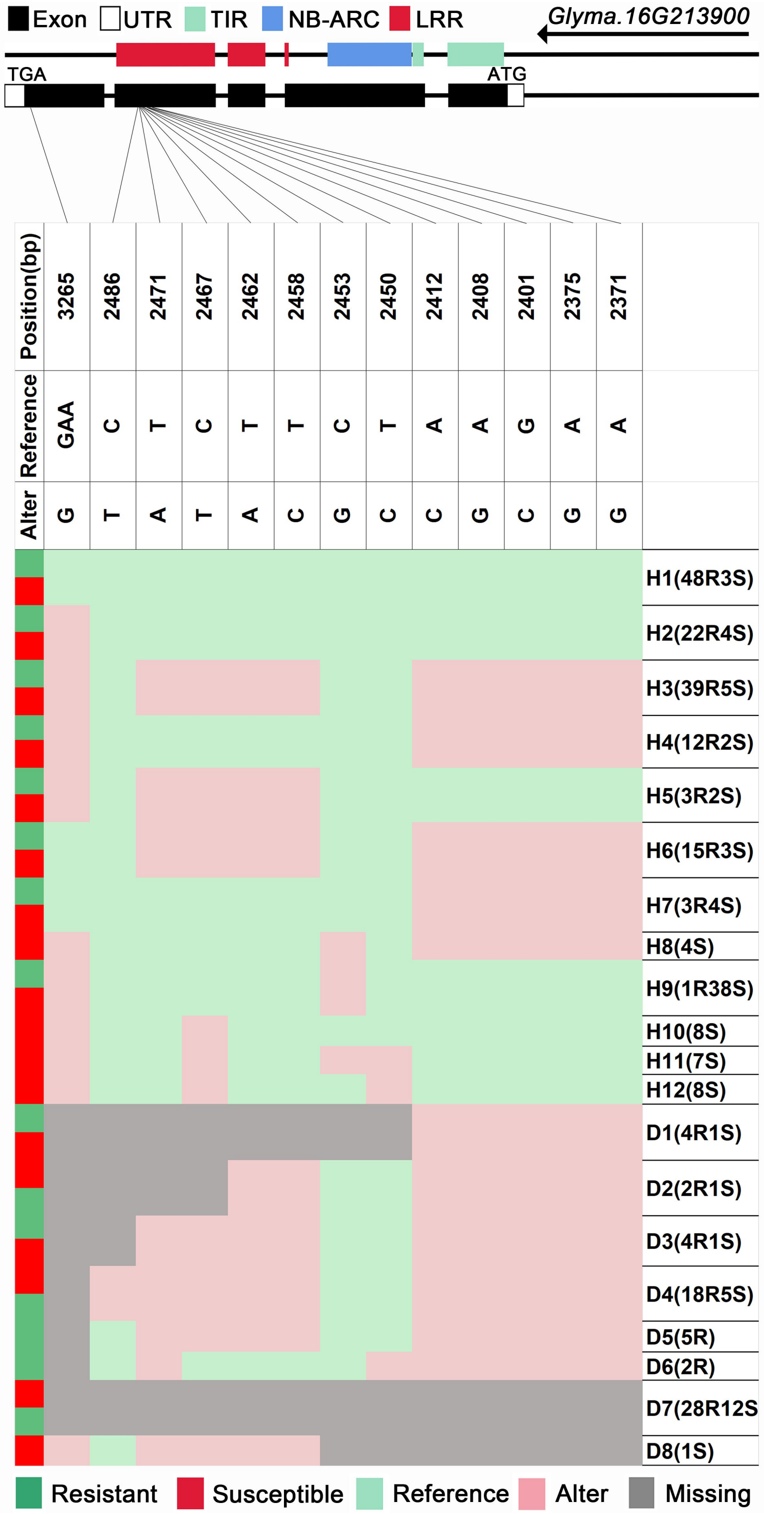

Supplementary Figure 5. Haplotype analysis of *Glyma.16g213900*. H:haplotype; R: Resistant; S: Susceptible; D1:Deletion type 1; TIR:Toll/interleukin-1 receptor; NBS:nucleotide-binding site. LRR:leucine-rich repeat domin.

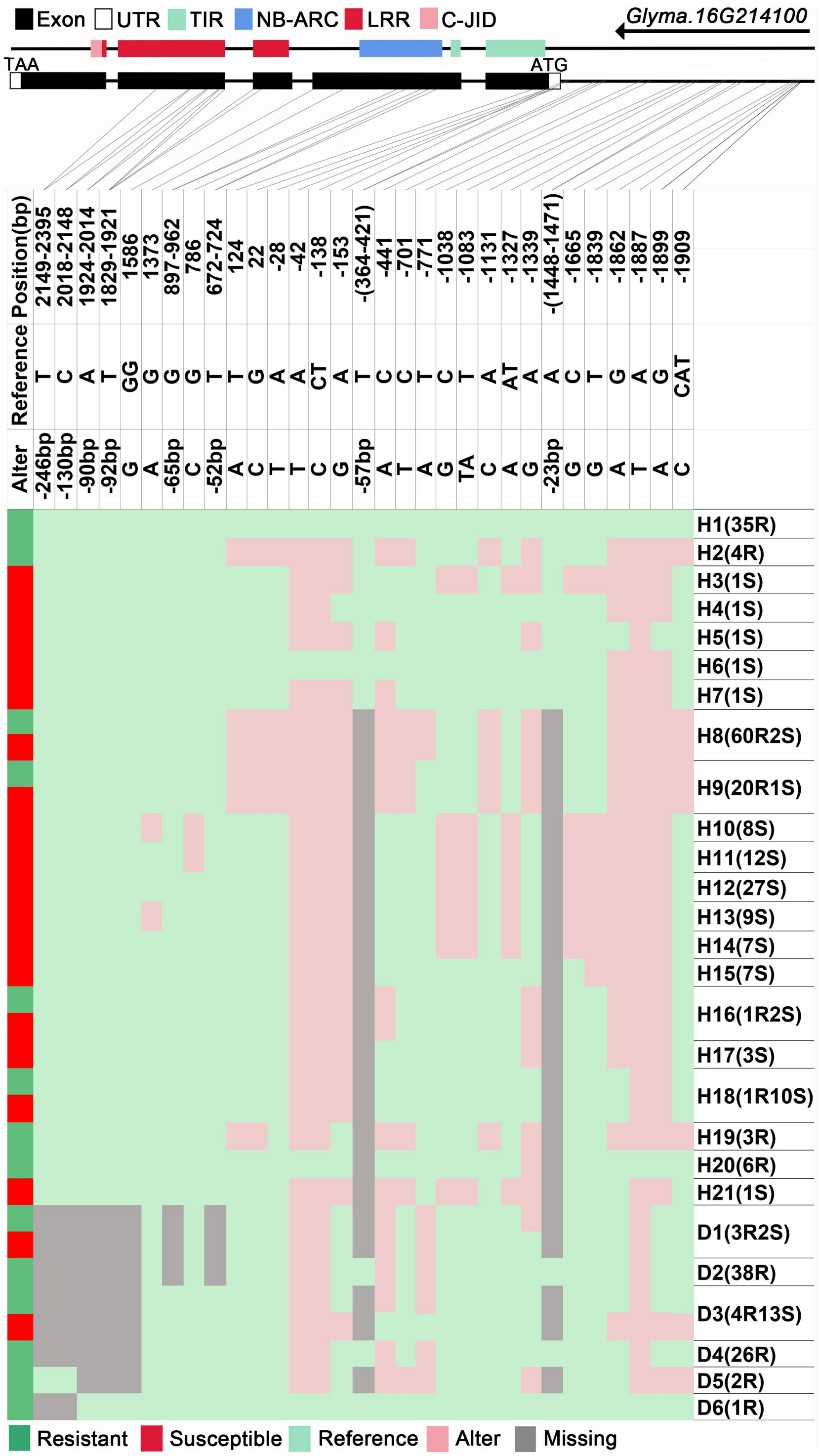

Supplementary Figure 6. Haplotype analysis of *Glyma.16g214100*. H: haplotype; R: Resistant; S: Susceptible; D1: Deletion type 1; TIR: Toll/interleukin-1 receptor; NBS: nucleotide-binding site; LRR: leucine-rich repeat domin; C-JID: C-terminal jelly roll/Ig-like domain.

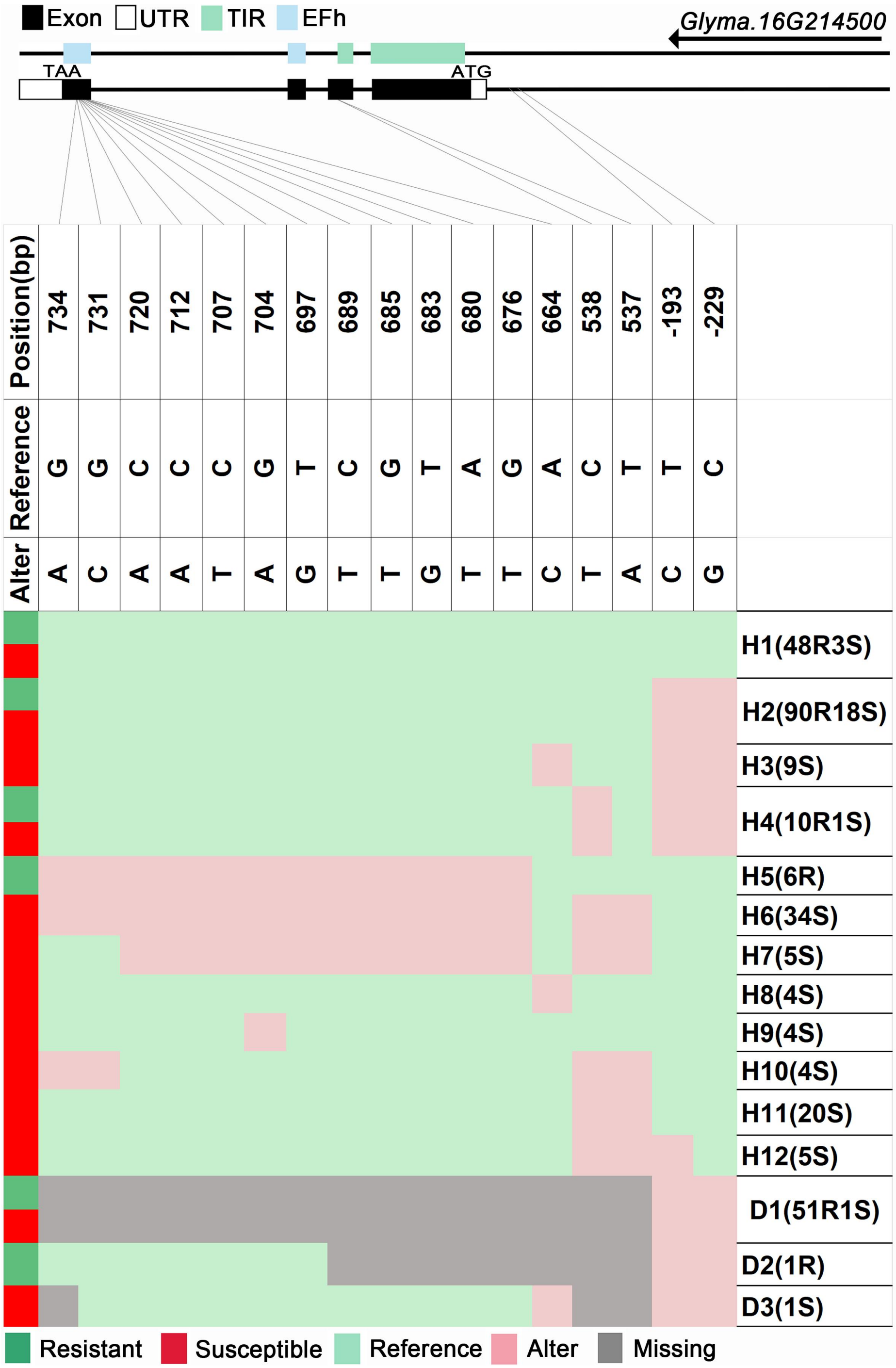

Supplementary Figure 7. Haplotype analysis of *Glyma.16g214500*. H: haplotype; R: Resistant; S: Susceptible; D1: Deletion type 1; TIR:Toll/interleukin-1 receptor; EFh: EF-hand domain.

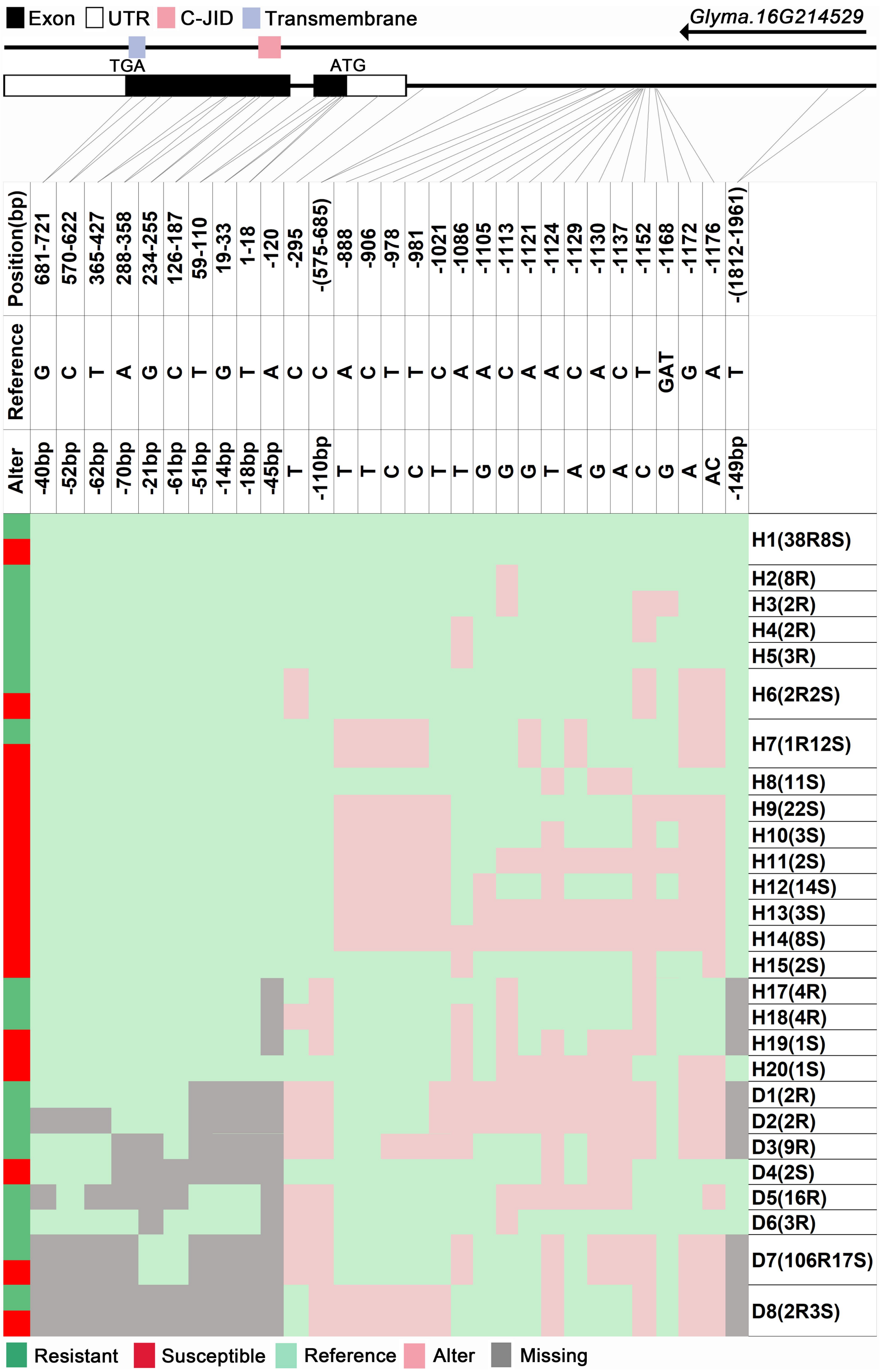

Supplementary Figure 8. Haplotype analysis of *Glyma.16g214529*. H: haplotype; R: Resistant; S: Susceptible; D1: Deletion type 1; C-JID: C-terminal jelly roll/Ig-like domain; Transmembrane: Transmembrane domain.

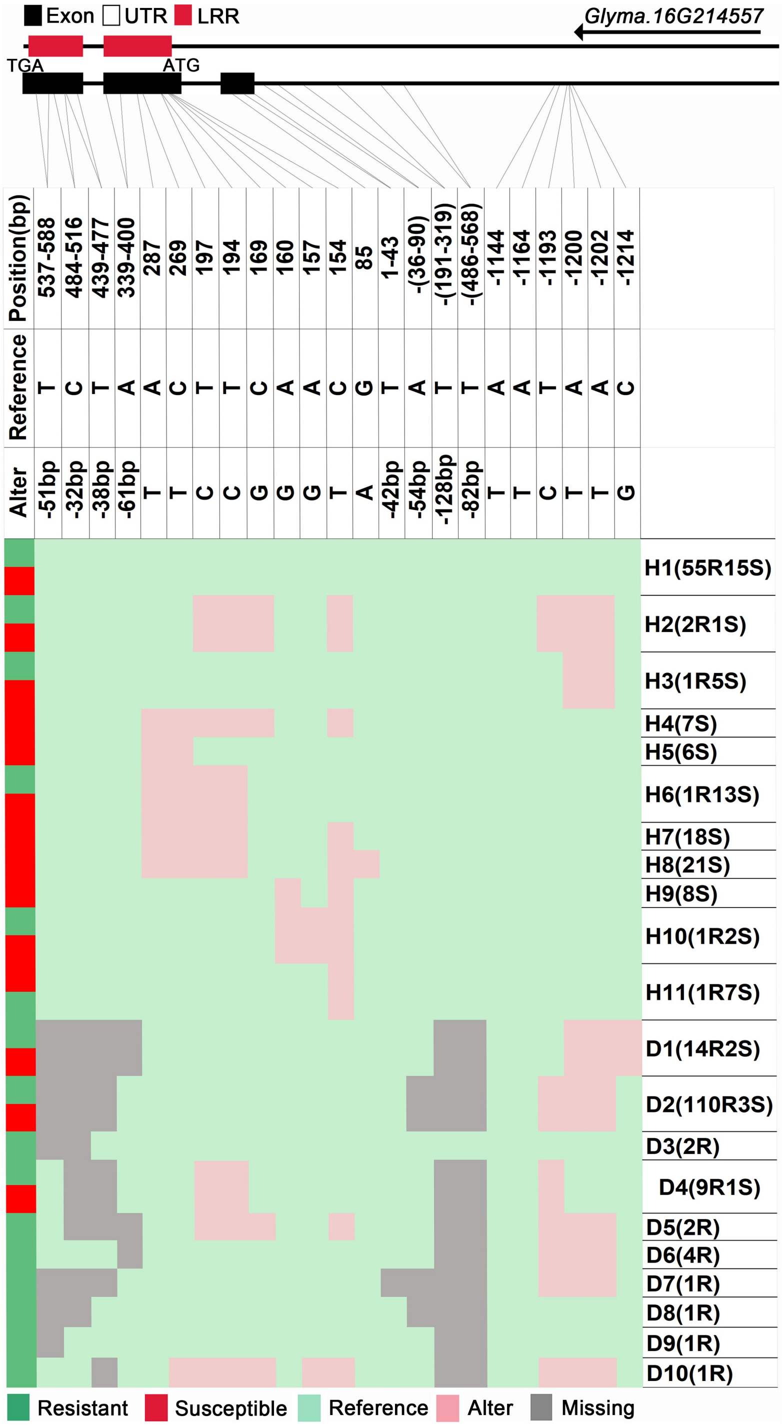

Supplementary Figure 9. Haplotype analysis of *Glyma.16g214557*. H: haplotype; R: Resistant; S: Susceptible; D1: Deletion type 1; LRR: leucine-rich repeat domin.

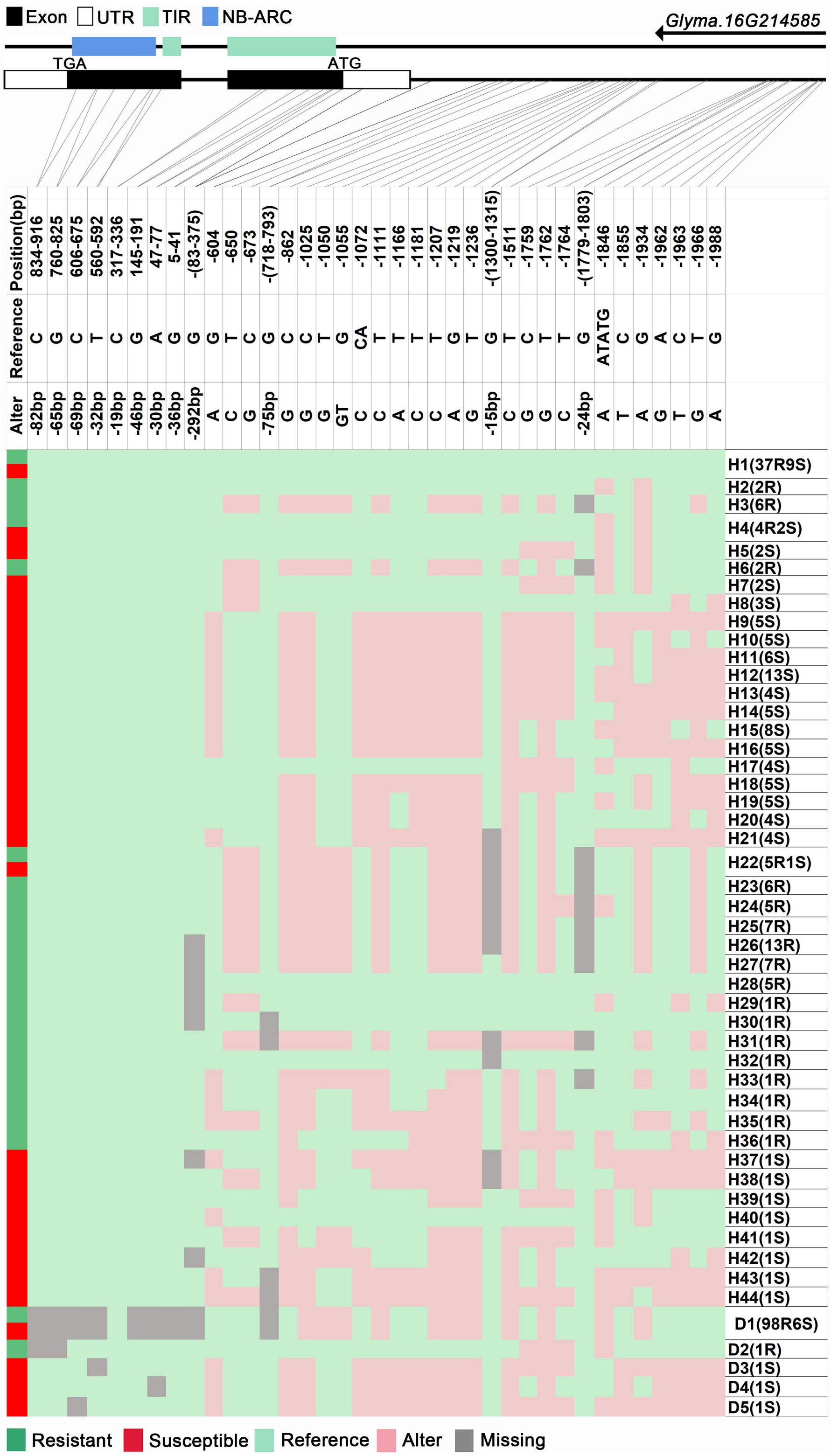

Supplementary Figure 10. Haplotype analysis of *Glyma.16g214585*. H: haplotype; R: Resistant; S: Susceptible; D1: Deletion type 1; TIR: Toll/interleukin-1 receptor; NBS: nucleotide-binding site.

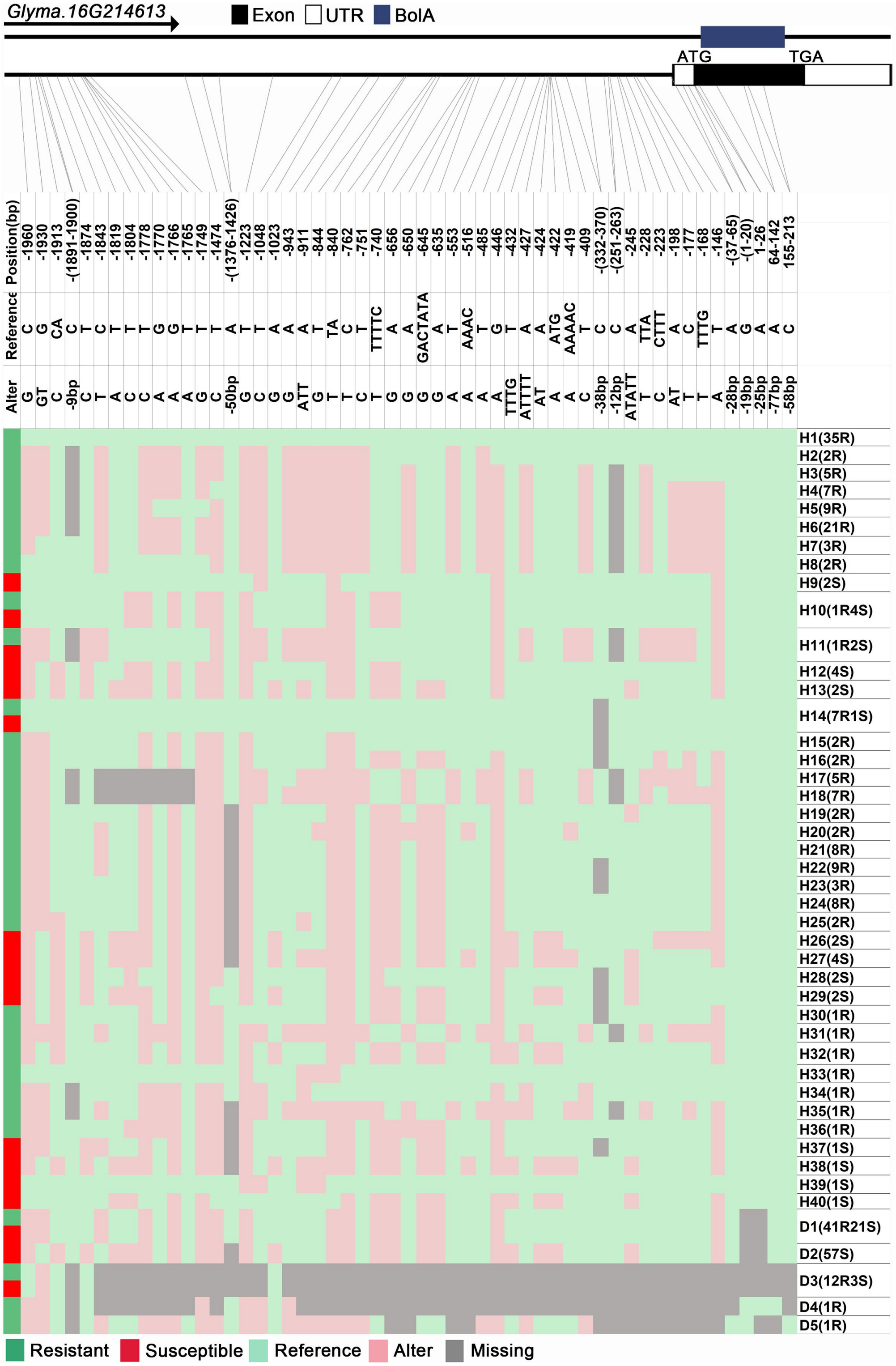

Supplementary Figure 11. Haplotype analysis of *Glyma.16g214613*. H: haplotype; R: Resistant; S: Susceptible; D1: Deletion type 1; BolA: BolA-like protein 2.

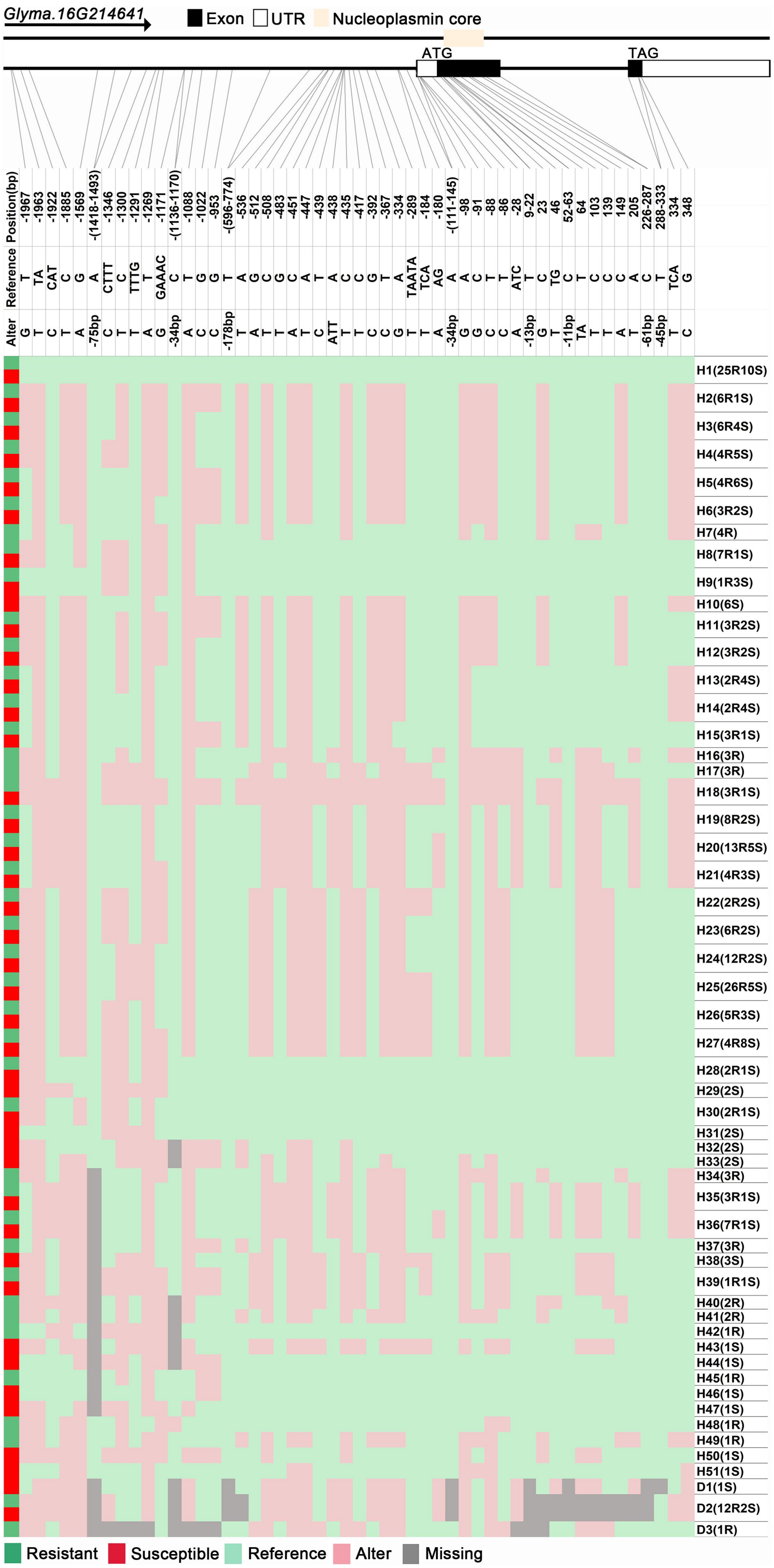

Supplementary Figure 12. Haplotype analysis of *Glyma.16g214641*. H: haplotype; R: Resistant; S: Susceptible; D1: Deletion type 1; Nucleoplasmin core: Nucleoplasmin core domain.

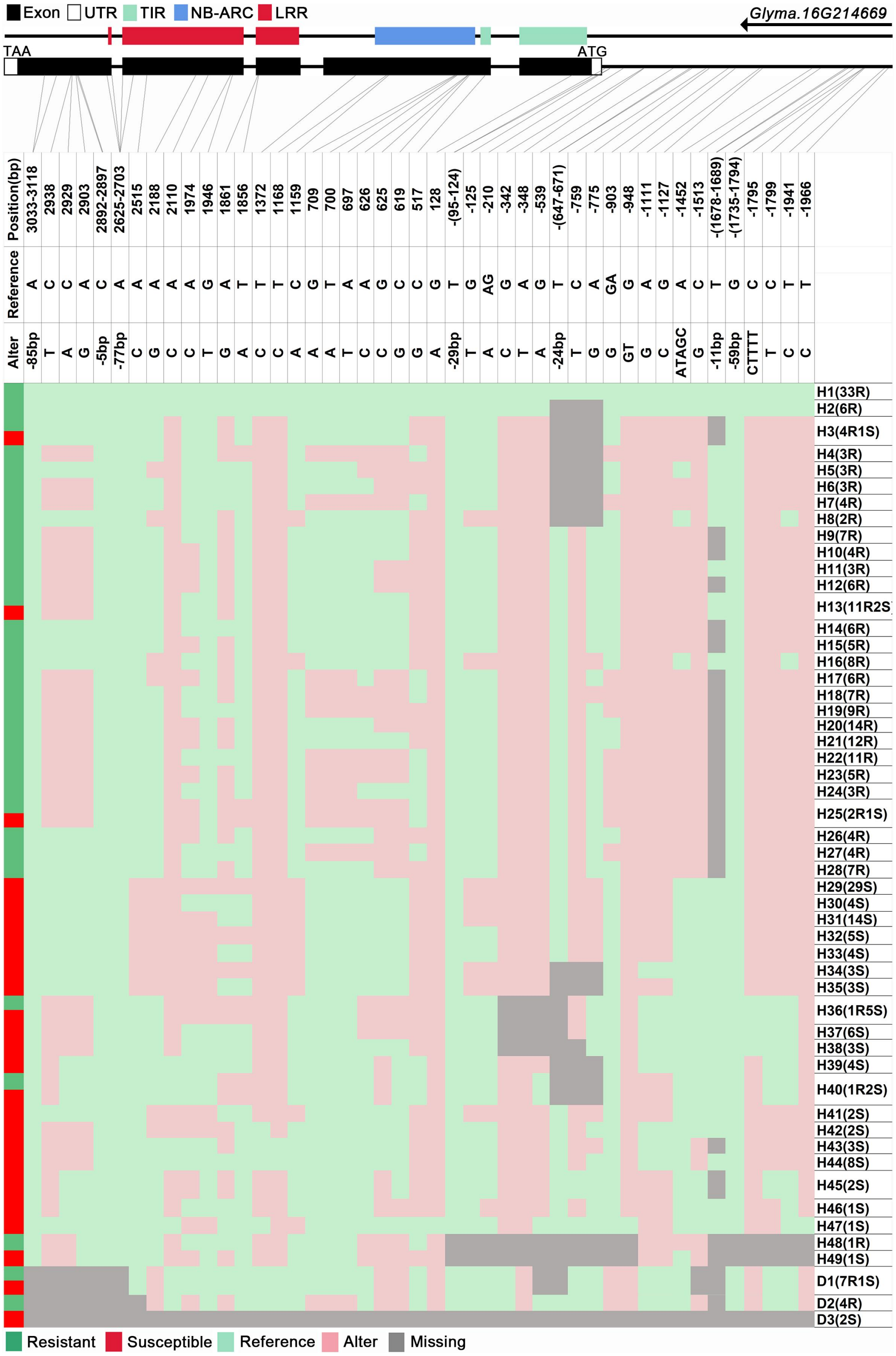

Supplementary Figure 13. Haplotype analysis of *Glyma.16g214669*. H:haplotype; R: Resistant; S: Susceptible; D1: Deletion type 1; TIR: Toll/interleukin-1 receptor; NBS: nucleotide-binding site; LRR: leucine-rich repeat domin.

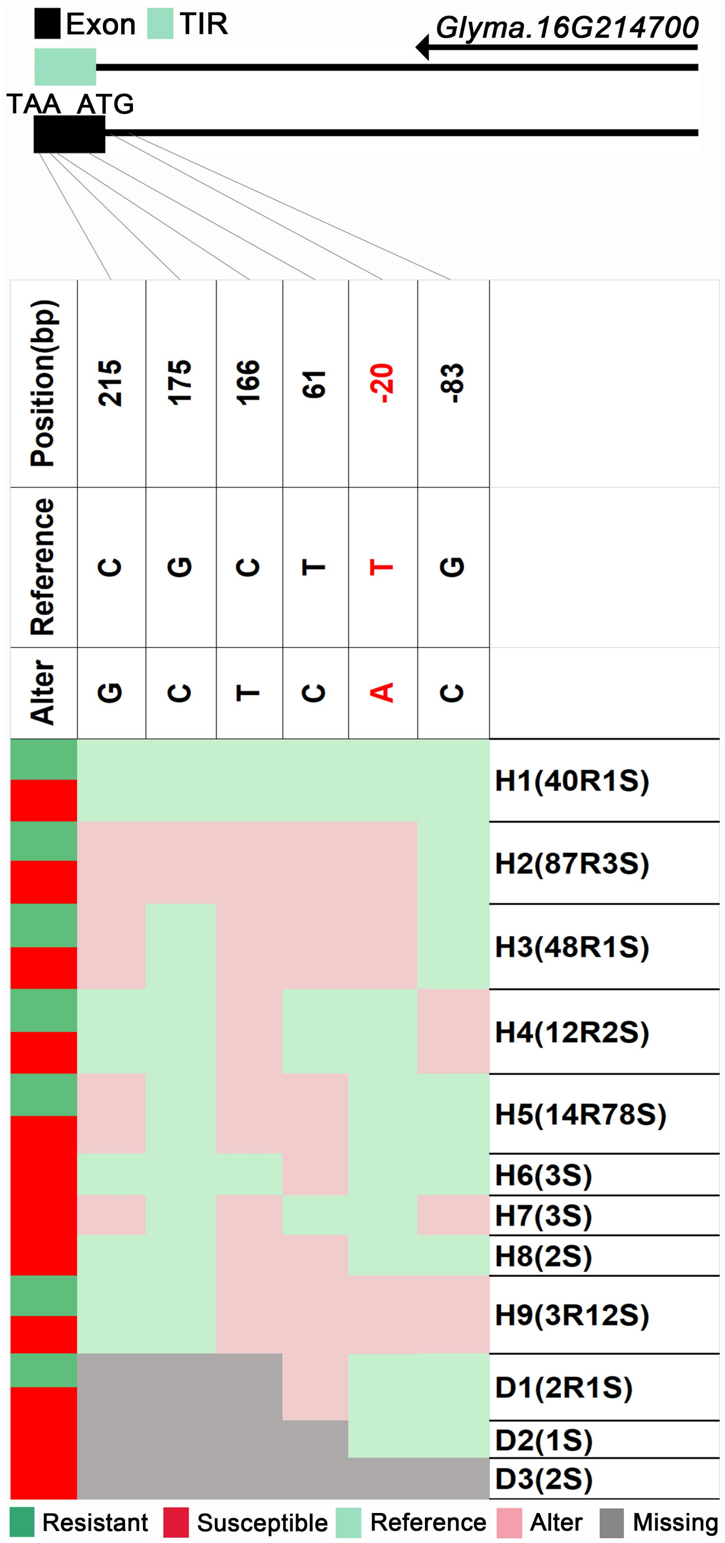

Supplementary Figure 14. Haplotype analysis of *Glyma.16g214700*. H:haplotype; R: Resistant; S: Susceptible; D1: Deletion type 1; TIR: Toll/interleukin-1 receptor.

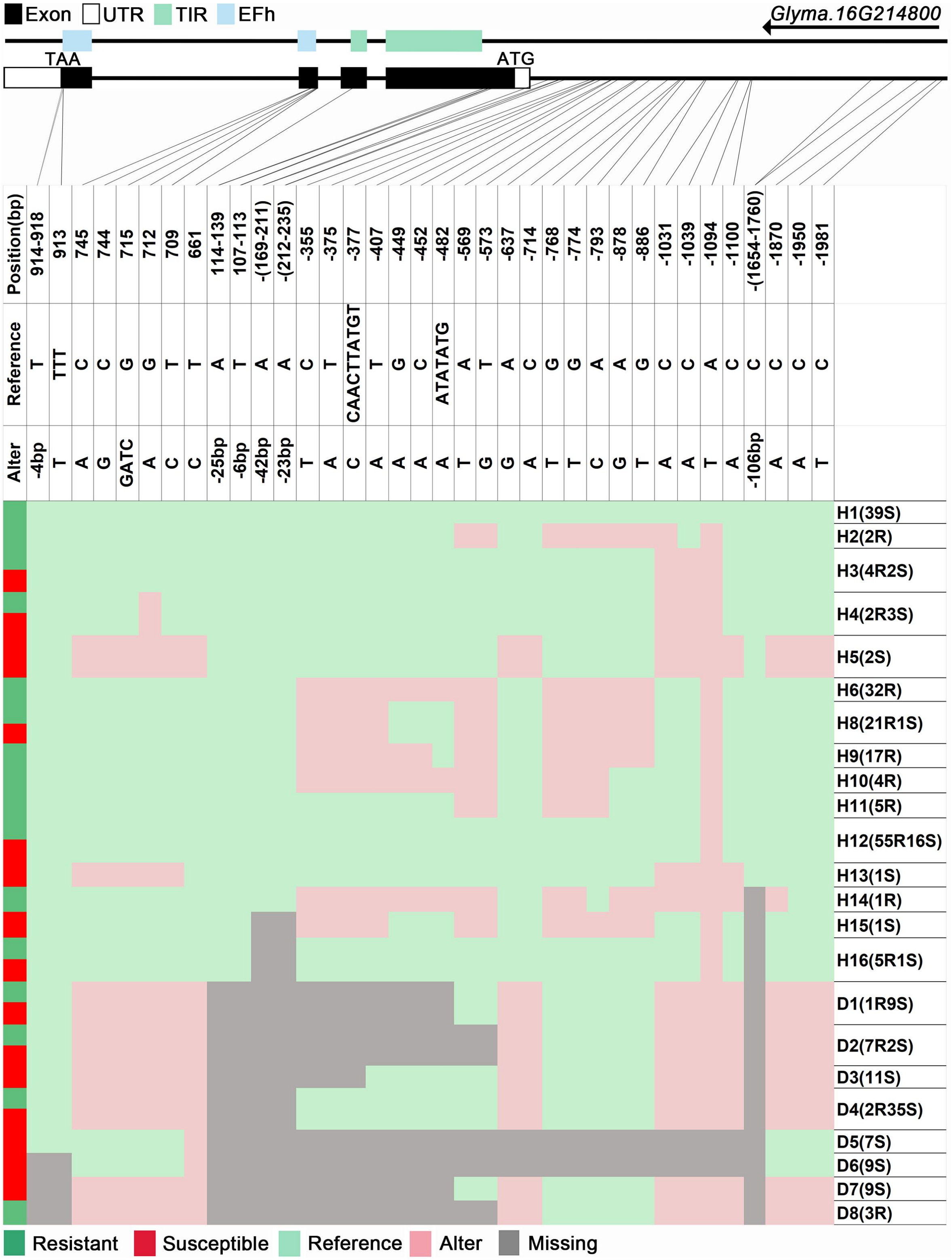

Supplementary Figure 15. Haplotype analysis of *Glyma.16g214800*. H: haplotype; R: Resistant; S: Susceptible; D1: Deletion type 1; TIR:Toll/interleukin-1 receptor; EFh: EF-hand domain.

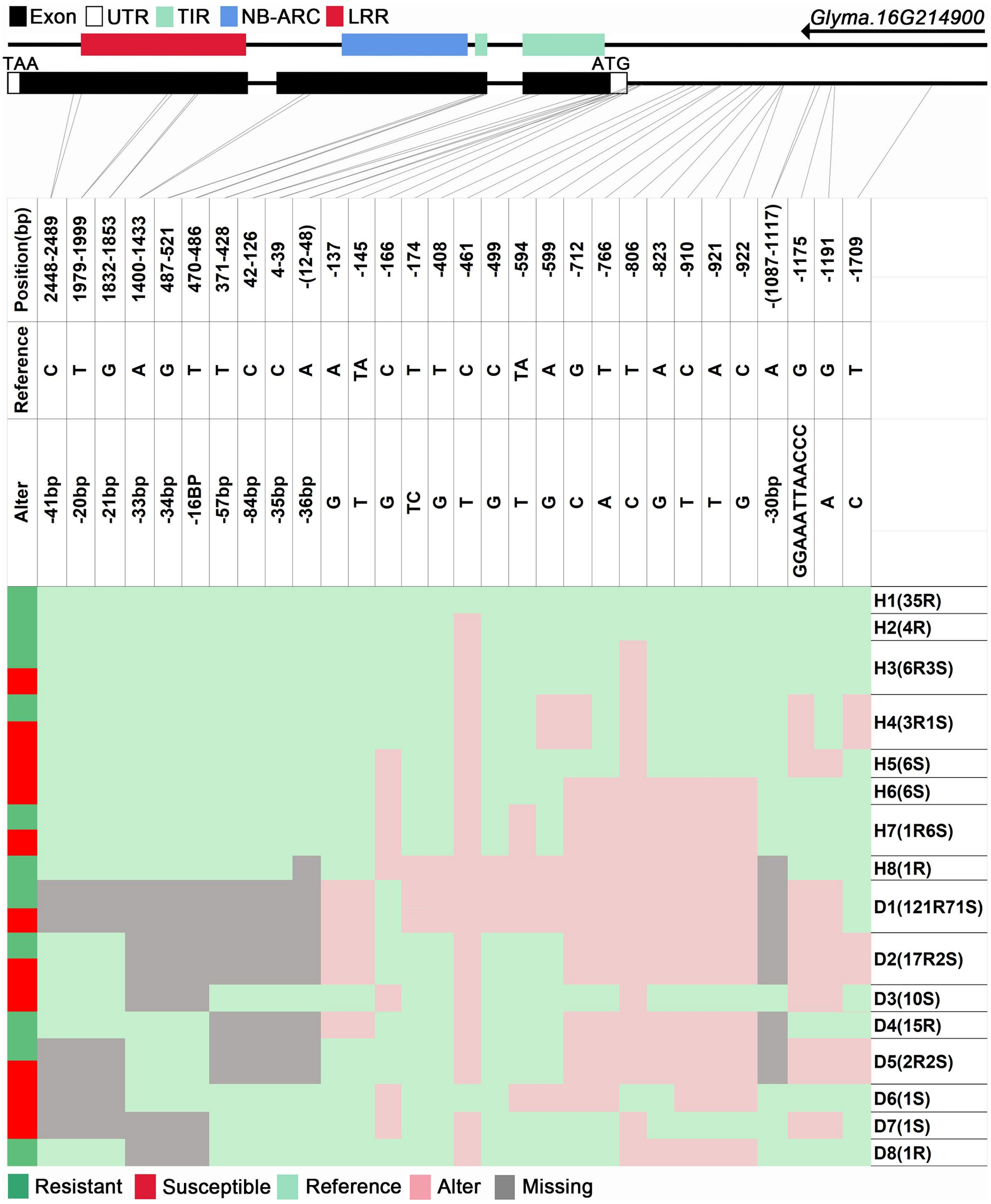

Supplementary Figure 16. Haplotype analysis of *Glyma.16g214900*. H: haplotype; R: Resistant; S: Susceptible; D1: Deletion type 1; TIR: Toll/interleukin-1 receptor; NBS: nucleotide-binding site; LRR: leucine-rich repeat domin.

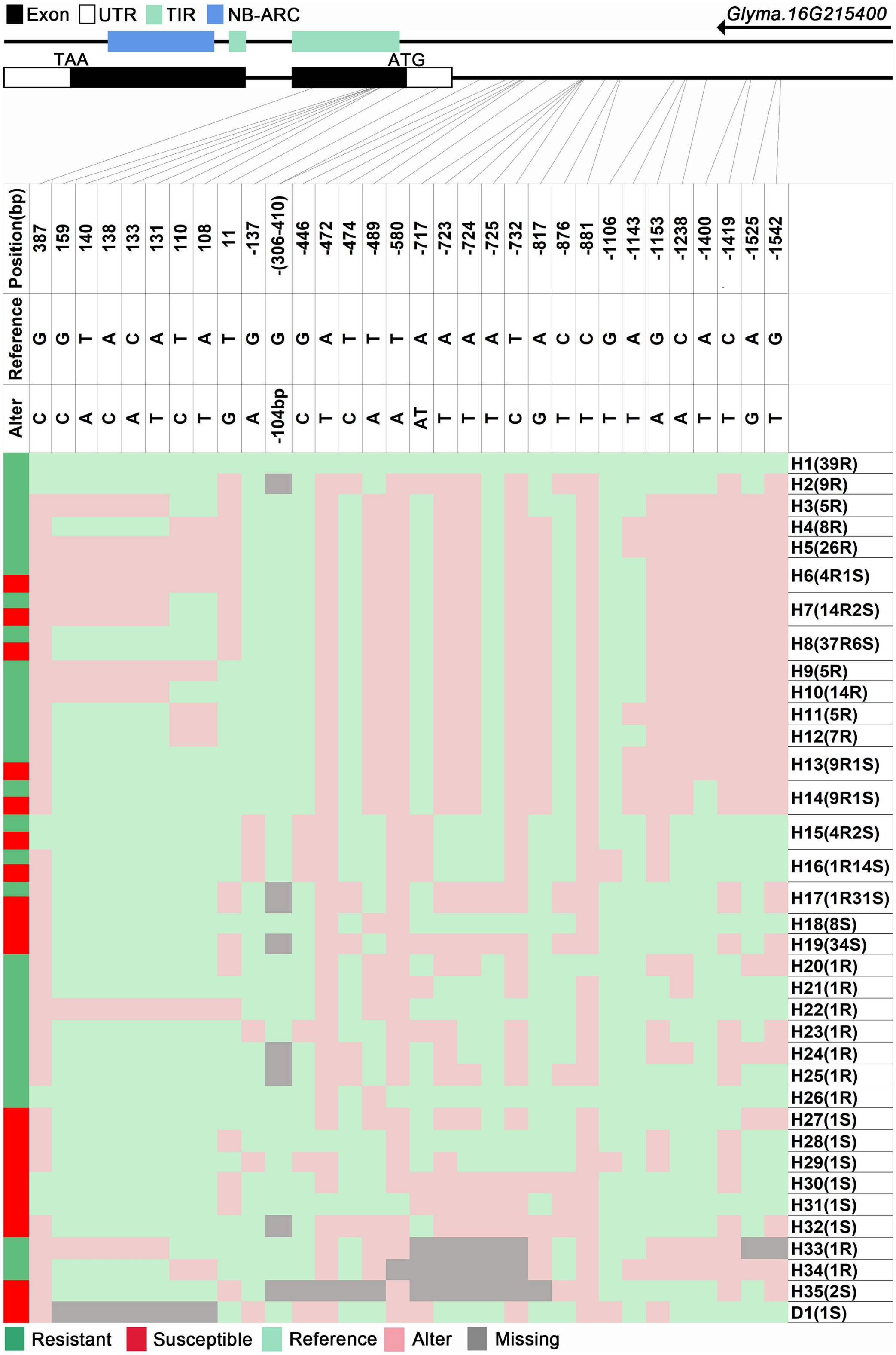

Supplementary Figure 17. Haplotype analysis of *Glyma.16g215400*. H: haplotype; R: Resistant; S: Susceptible; D1: Deletion type 1; TIR: Toll/interleukin-1 receptor; NBS: nucleotide-binding site.

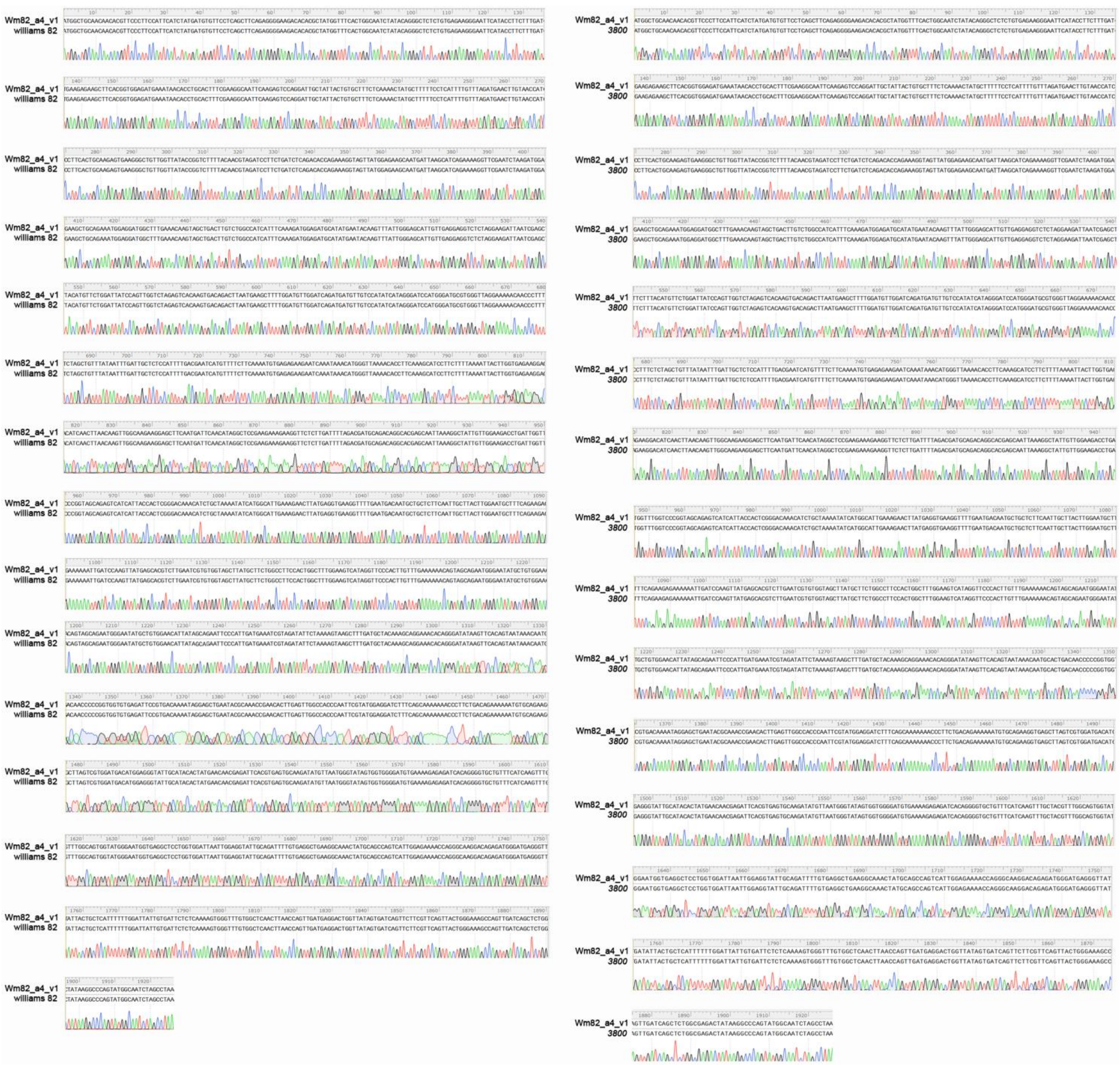

Supplementary Figure 18. Sequencing peaks of the *Glyma.16G214300* from lines *Williams 82* and *3800*. Wm82_a4_v1: reference genome annotation Wm82.a4.v1 version. *Williams 82*: Control group. *3800*: EMS-induced Williams 82 mutant, the *Glyma.16G213800* is mutated.

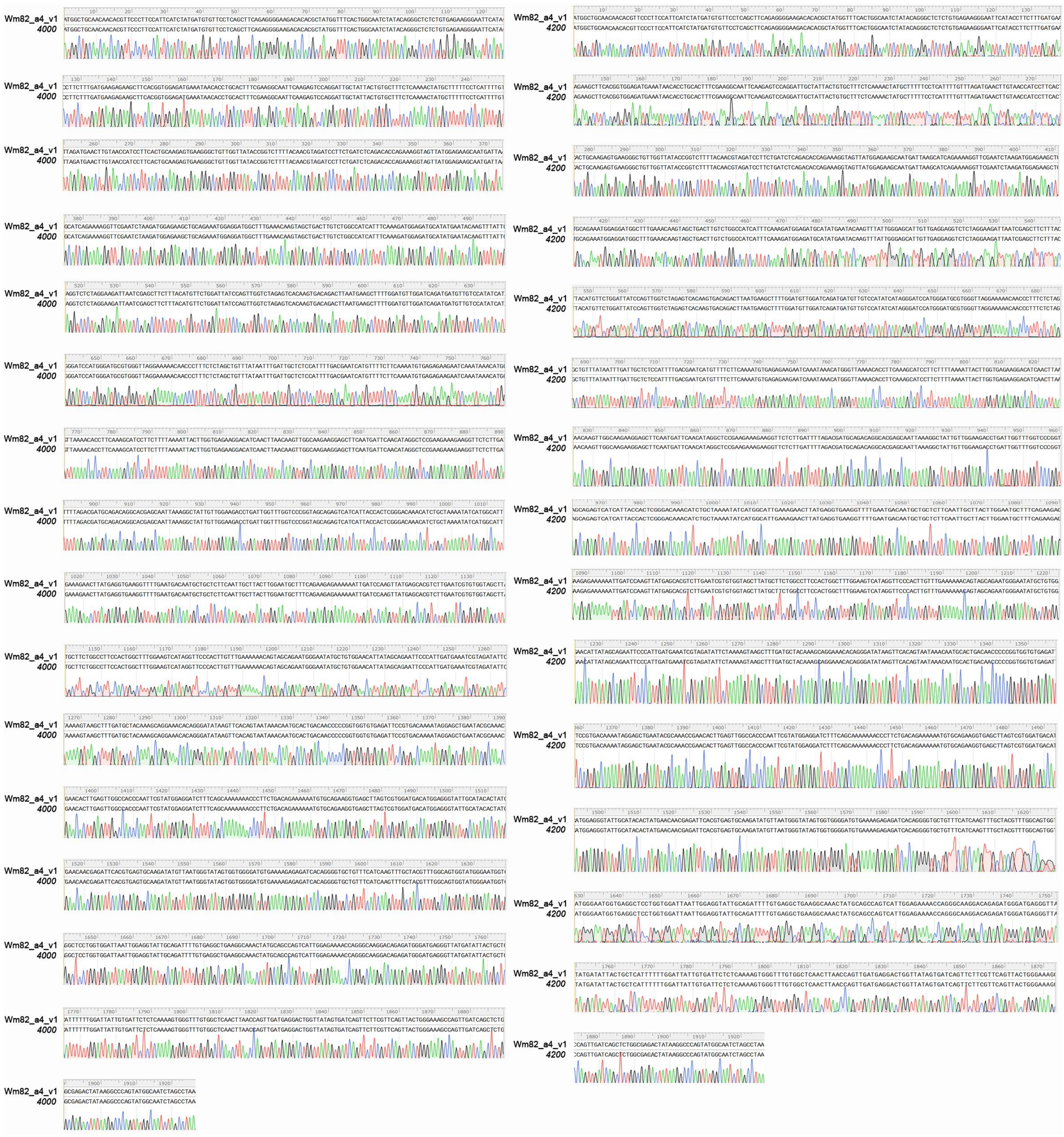

Supplementary Figure 19. Sequencing peaks of the *Glyma.16G214300* from lines *4000* and *4200*. Wm82_a4_v1: reference genome annotation Wm82.a4.v1 version. *4000*: EMS-induced Williams 82 mutant, the *Glyma.16G214000* is mutated. *4200*: EMS-induced Williams 82 mutant, the *Glyma.16G214200* is mutated.

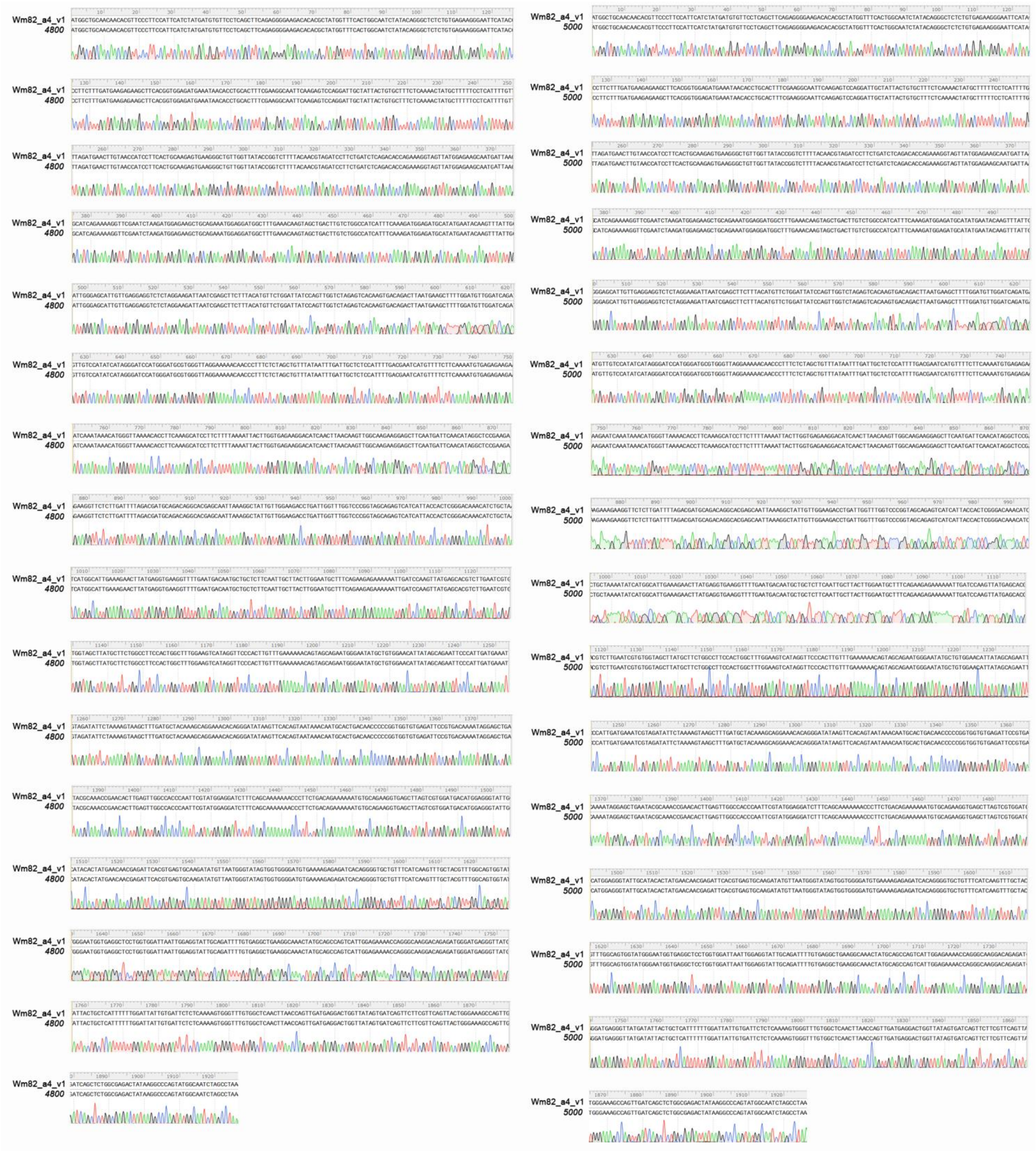

Supplementary Figure 20. Sequencing peaks of the *Glyma.16G214300* from lines *4800* and *5000*. Wm82_a4_v1: reference genome annotation Wm82.a4.v1 version. *4800*: EMS-induced Williams 82 mutant, the *Glyma.16G214800* is mutated. *5000*: EMS-induced Williams 82 mutant, the *Glyma.16G215000* is mutated.

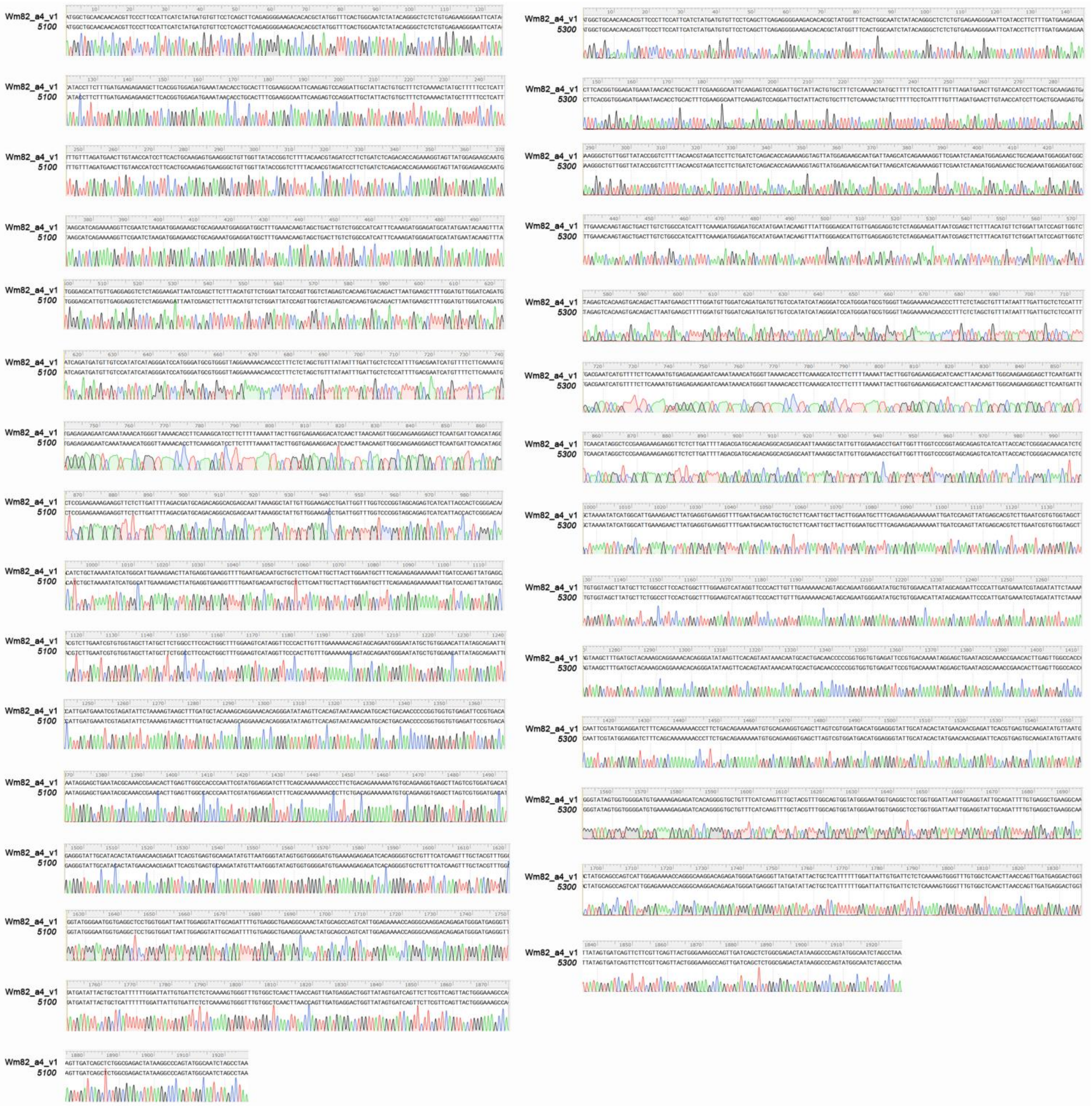

Supplementary Figure 21. Sequencing peaks of the *Glyma.16G214300* from lines *5100* and *5300*. Wm82_a4_v1: reference genome annotation Wm82.a4.v1 version. *5100*: EMS-induced Williams 82 mutant, the *Glyma.16G215100* is mutated. *5300*: EMS-induced Williams 82 mutant, the *Glyma.16G215300* is mutated.

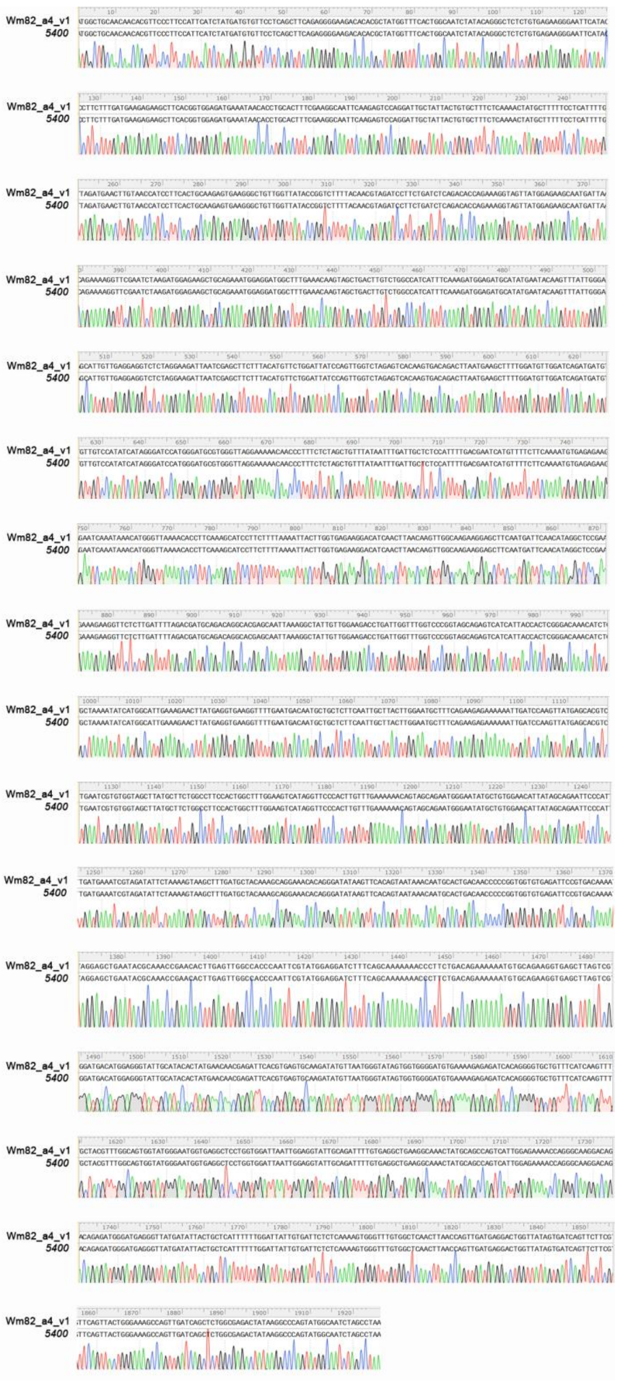

Supplementary Figure 22. Sequencing peaks of the *Glyma.16G214300* from lines *5400*. Wm82_a4_v1: reference genome annotation Wm82.a4.v1 version. *5400*: EMS-induced Williams 82 mutant, the *Glyma.16G215400* is mutated.

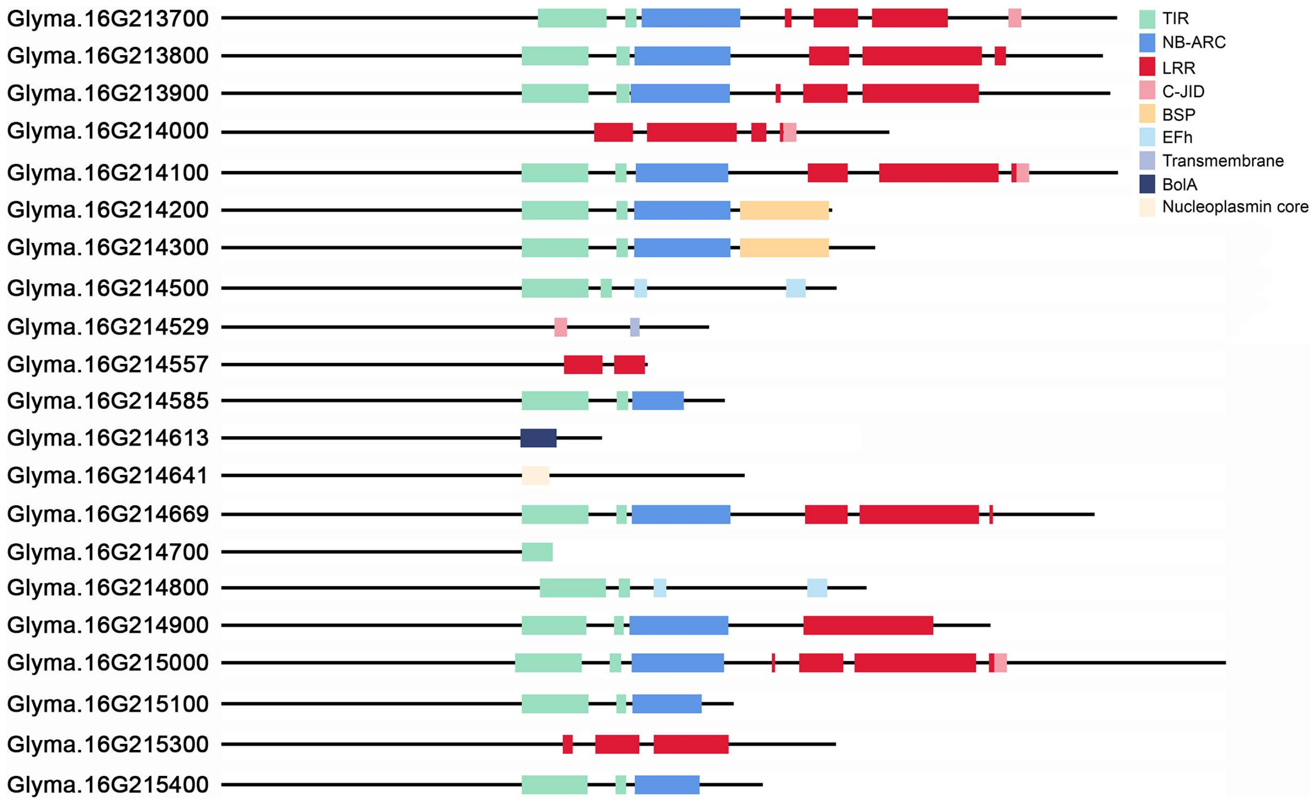

Supplementary Figure 23. Domain analysis of candidate genes. TIR: Toll/interleukin-1 receptor; NBS: nucleotide-binding site; LRR: leucine-rich repeat domin; C-JID: C-terminal jelly roll/Ig-like domain; BSP: Basic Secretory Proteins; EFh: EF-hand domain; Transmembrane: Transmembrane domain; BolA: BolA-like protein 2; Nucleoplasmin core: Nucleoplasmin core domain.

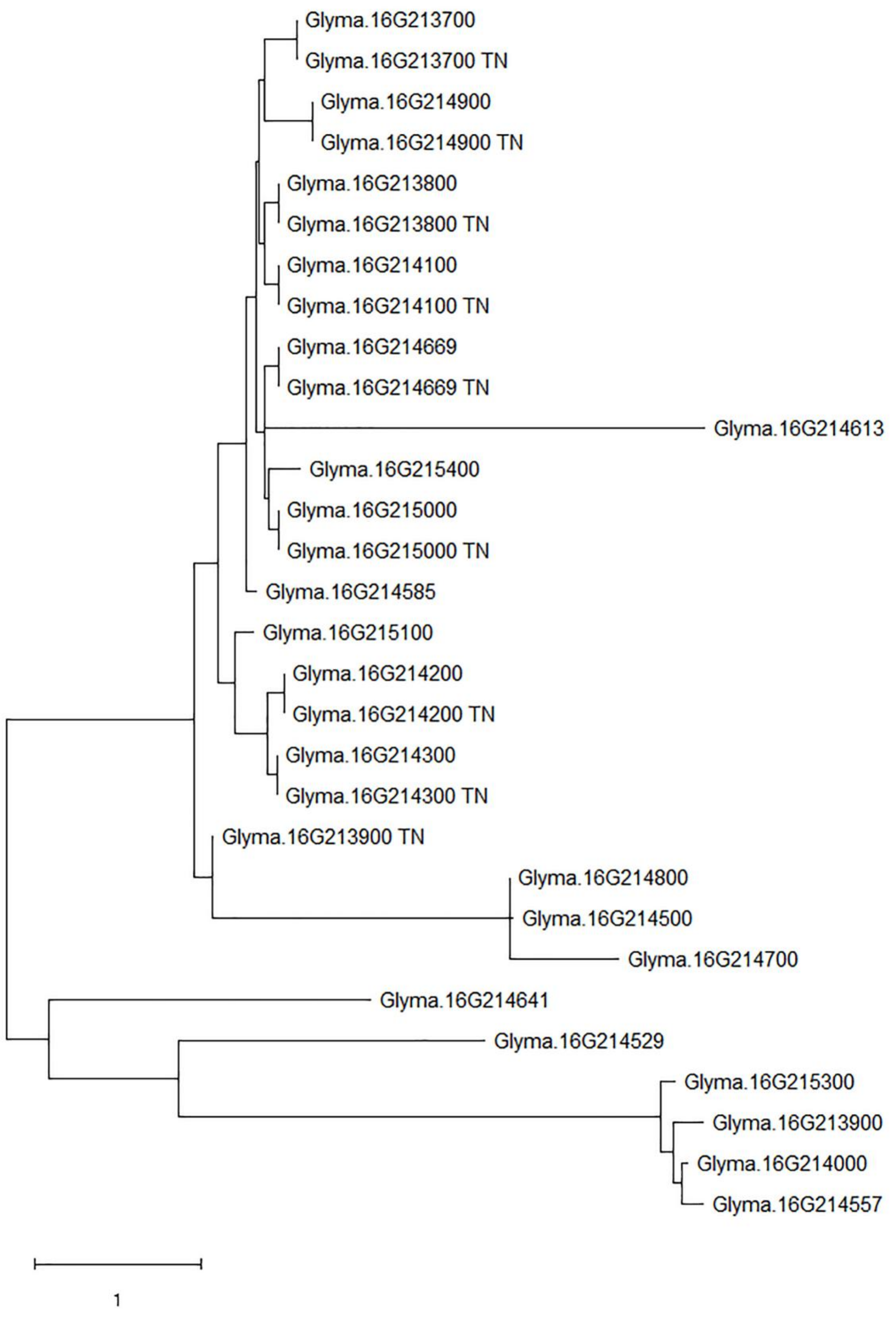

Supplementary Figure 24. Phylogenetic analysis of complete and truncated proteins encoded by candidate genes. TN: Proteins exclusively harboring Toll/interleukin-1 receptor (TIR) and nucleotide-binding site (NBS) domains.
